# Rescue of dead MnO_2_ for stable electrolytic Zn–Mn redox-flow battery: a metric of mediated and catalytic kinetics

**DOI:** 10.1093/nsr/nwae230

**Published:** 2024-07-03

**Authors:** Qi Wang, Wanhai Zhou, Yanyan Zhang, Hongrun Jin, Xinran Li, Tengsheng Zhang, Boya Wang, Ruizheng Zhao, Junwei Zhang, Wei Li, Yu Qiao, Chuankun Jia, Dongyuan Zhao, Dongliang Chao

**Affiliations:** Laboratory of Advanced Materials, Shanghai Key Laboratory of Molecular Catalysis and Innovative Materials, State Key Laboratory of Molecular Engineering of Polymers, College of Chemistry and Materials, Fudan University, Shanghai 200433, China; Laboratory of Advanced Materials, Shanghai Key Laboratory of Molecular Catalysis and Innovative Materials, State Key Laboratory of Molecular Engineering of Polymers, College of Chemistry and Materials, Fudan University, Shanghai 200433, China; Laboratory of Advanced Materials, Shanghai Key Laboratory of Molecular Catalysis and Innovative Materials, State Key Laboratory of Molecular Engineering of Polymers, College of Chemistry and Materials, Fudan University, Shanghai 200433, China; Laboratory of Advanced Materials, Shanghai Key Laboratory of Molecular Catalysis and Innovative Materials, State Key Laboratory of Molecular Engineering of Polymers, College of Chemistry and Materials, Fudan University, Shanghai 200433, China; Laboratory of Advanced Materials, Shanghai Key Laboratory of Molecular Catalysis and Innovative Materials, State Key Laboratory of Molecular Engineering of Polymers, College of Chemistry and Materials, Fudan University, Shanghai 200433, China; Laboratory of Advanced Materials, Shanghai Key Laboratory of Molecular Catalysis and Innovative Materials, State Key Laboratory of Molecular Engineering of Polymers, College of Chemistry and Materials, Fudan University, Shanghai 200433, China; Laboratory of Advanced Materials, Shanghai Key Laboratory of Molecular Catalysis and Innovative Materials, State Key Laboratory of Molecular Engineering of Polymers, College of Chemistry and Materials, Fudan University, Shanghai 200433, China; Laboratory of Advanced Materials, Shanghai Key Laboratory of Molecular Catalysis and Innovative Materials, State Key Laboratory of Molecular Engineering of Polymers, College of Chemistry and Materials, Fudan University, Shanghai 200433, China; Laboratory of Advanced Materials, Shanghai Key Laboratory of Molecular Catalysis and Innovative Materials, State Key Laboratory of Molecular Engineering of Polymers, College of Chemistry and Materials, Fudan University, Shanghai 200433, China; Laboratory of Advanced Materials, Shanghai Key Laboratory of Molecular Catalysis and Innovative Materials, State Key Laboratory of Molecular Engineering of Polymers, College of Chemistry and Materials, Fudan University, Shanghai 200433, China; State Key Laboratory of Physical Chemistry of Solid Surfaces, Collaborative Innovation Center of Chemistry for Energy Materials (iChEM), Department of Chemistry, College of Chemistry and Chemical Engineering, Xiamen University, Xiamen 361005, China; Institute of Energy Storage Technology, College of Materials Science and Engineering, Changsha University of Science & Technology, Changsha 410114, China; Laboratory of Advanced Materials, Shanghai Key Laboratory of Molecular Catalysis and Innovative Materials, State Key Laboratory of Molecular Engineering of Polymers, College of Chemistry and Materials, Fudan University, Shanghai 200433, China; Laboratory of Advanced Materials, Shanghai Key Laboratory of Molecular Catalysis and Innovative Materials, State Key Laboratory of Molecular Engineering of Polymers, College of Chemistry and Materials, Fudan University, Shanghai 200433, China

**Keywords:** aqueous batteries, high areal capacity, dead MnO_2_, redox mediator, catalysed kinetics

## Abstract

The virtues of electrolytic MnO_2_ aqueous batteries are high theoretical energy density, affordability and safety. However, the continuous dead MnO_2_ and unstable Mn^2+^/MnO_2_ electrolysis pose challenges to the practical output energy and lifespan. Herein, we demonstrate bifunctional cationic redox mediation and catalysis kinetics metrics to rescue dead MnO_2_ and construct a stable and fast electrolytic Zn–Mn redox-flow battery (eZMRFB). Spectroscopic characterizations and electrochemical evaluation reveal the superior mediation kinetics of a cationic Fe^2+^ redox mediator compared with the anionic ones (e.g. I^–^ and Br^–^), thus eliminating dead MnO_2_ effectively. With intensified oxygen vacancies, density functional theory simulations of the reaction pathways further verify the concomitant Fe-catalysed Mn^2+^/MnO_2_ electrolysis kinetics via charge delocalization and activated O 2p electron states, boosting its rate capability. As a result, the elaborated eZMRFB achieves a coulombic efficiency of nearly 100%, ultra-high areal capacity of 80 mAh cm^–2^, rate capability of 20 C and a long lifespan of 2500 cycles. This work may advance high-energy aqueous batteries to next-generation scalable energy storage.

## Teaser

A metric of mediated kinetics and the concomitant Fe-catalysed Mn^2+^/MnO_2_ electrolysis kinetics to rescue dead MnO_2_ for stable Zn–Mn redox-flow battery with a high areal capacity of 80 mAh cm^–2^.

## INTRODUCTION

Advanced aqueous batteries possess features of high safety, affordability and eco-friendliness, which are the urgent requirements for the electrification process [1–3]. Among them, electrolytic Zn–Mn aqueous batteries are attractive due to their bi-electronic (616 mAh g^–1^) mechanism [4–6], high theoretical energy density of >700 Wh kg^–1^, high operation voltage of 1.9–2.9 V and low cost of <US${\$}$10 per kWh [7–9]. However, owing to the low conductivity of MnO_2_ (∼10^–6^ S cm^–1^) and high mechanical stress during the solid-to-liquid conversion [10–14], the formation of a thick deposition layer inevitably triggers the generation of dead MnO_2_, i.e. the exfoliated or incompletely dissolved MnO_2_. The limited mass loading should result in unsatisfactory areal capacity, which is deemed indispensable for high practical energy output [15–17]. Simultaneously, sluggish MnO_2_/Mn^2+^ electrolysis kinetics at high areal capacities leading to large overpotential would circularly cause anabatic dead MnO_2_. The dead MnO_2_ should accumulate with impeded ion transportation and/or exfoliate with drastic capacity loss in the prolonged cycles, thus resulting in poor rate capability and rapid battery failure [18–20].

To date, in order to stabilize the Mn^2+^/MnO_2_ electrolytic process, various approaches, including element doping [21,22], pH regulation [4,23–26] and redox mediation [27–31], have been explored. For example, Ni and Co doping was applied to regulate the electronic structure and defect of MnO_2_ [21,22], which essentially enhanced the electronic and ionic conductivity and achieved an areal capacity of 10 mAh cm^–2^ [22]. Adopting acetate electrolytes can inhibit the formation of Mn^3+^ via the coordinated effect of acetate on Mn^2+^ and pH stabilization, thereby preventing the occurrence of the disproportionated dead MnO_2_ [24,25]. This expands the Zn–MnO_2_ aqueous battery to a substantial areal capacity of 20 mAh cm^–2^, with a limited lifespan of 30 cycles [26]. The I^–^ and Br^–^ anionic redox mediators (RMs) have been employed to offer an additional charge-transfer route beyond the localized interface [32–34] and recover ‘lost’ capacity from exfoliated dead MnO_2_ [27–31]. Recently, Lu *et al.* achieved a high areal capacity of ∼50 mAh cm^–2^ with 50 cycles in the I^–^-mediated neutral Zn–MnO_2_ aqueous battery through an acetate electrolyte [27]. The acetate-based neutral electrolytic Zn–Mn aqueous battery exhibits a working voltage of ∼1.4 V and energy efficiency (EE) of <80%, which are inferior to the acidic ones (∼2.0 V and 90% of EE) [30,35]. Be employing I^–^ and Br^–^ anionic RMs, Chen and co-workers achieved an areal capacity of 13.3 mAh cm^–2^ [30,35]. Due to the poor solubility of I_2_/Br_2_ and their shielding of the active MnO_2_ [28,36], it is rational that the dead MnO_2_ cannot be fully eliminated after cycles, especially at high loadings. More practically, considering the heavy proportion of the inactive cathodic current collector (66 mg cm^–2^ of 5-mm carbon felt), a high areal capacity of >50 mAh cm^–2^ is necessary for a promising energy output, as conservatively calculated in Fig. S1. Hence, in order to rescue the dead MnO_2_ and further enhance the areal capability, the kinetic metrics of the RM for the Mn^2+^/MnO_2_ electrolysis process need to be carefully evaluated, which would be essential, both currently and in the long run.

In this work, we demonstrate a bifunctional Fe^2+^ cationic strategy via mediation and catalysis-boosted kinetics to rescue dead MnO_2_. Fe^2+^/Fe^3+^ redox not only serves as an ideal RM with kinetic merit to fleetingly and effectively eliminate dead MnO_2_, but also intensifies oxygen vacancies to catalyse and accelerate the Mn^2+^/MnO_2_ electrolytic process intrinsically. Consequently, as a proof of concept, the designed Fe^2+^-mediated electrolytic Zn–Mn redox-flow battery (Fe–eZMRFB) exhibits nearly 100% of coulombic efficiency, record high areal capacity (80 mAh cm^–2^), excellent rate capability (without obvious capacity loss at 20 C, 2.5 mAh cm^–2^ and 90% retention at 1 C, 50 mAh cm^–2^) and a long lifespan (2500 cycles at 1 mAh cm^–2^ and 100 cycles at 50 mAh cm^–2^). Through concomitantly tuning the mediation and catalysis kinetics, our results provide a new avenue to feasible energetic aqueous batteries and may be of immediate benefit for practical large-scale energy storage.

## RESULTS AND DISCUSSION

### Thermodynamic and kinetics metrics of RM design

**Figure 1.**
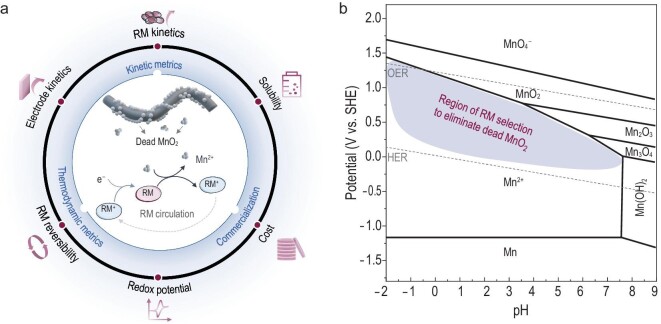
Metrics of RM design for the elimination of dead MnO_2_. (a) Critical parameters that affect the performance of the MnO_2_ electrolytic process. (b) Suitable redox-potential region in Mn-H_2_O Pourbaix diagram for RM design.

**Figure 2.**
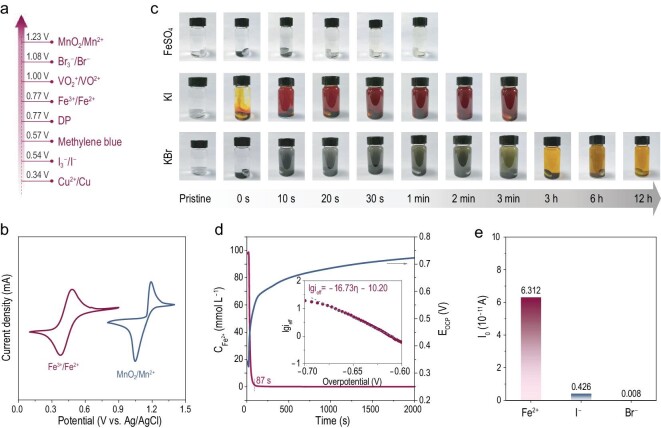
Thermodynamics and kinetics feasibility analyses of Fe^2+^ RM. (a) Redox potentials of various electrode materials. (b) CV curves of 0.05 M MnSO_4_ and 0.05 M FeSO_4_ in 0.5 M H_2_SO_4_ at 1 mV s^–1^. (c) Optical images of the suspended solid MnO_2_ eliminated by Fe^2+^, I^–^ and Br^–^ electrolytes at different durations. (d) Changes in the OCP and Fe^2+^ concentrations when MnO_2_ is mediated by Fe^2+^. The inset shows the changes in the reaction flux with overpotential and the fitting in terms of Butler–Volmer formulism. (e) RM reaction kinetics of the spontaneous chemical reaction between different RMs and MnO_2_.

RMs can facilitate the chemical/electrochemical reactions of solid reactants without physical contact with the current collector, thus extending the lifespan. To achieve effective elimination of dead MnO_2_, the chosen metrics of the ideal RMs are first summarized in Fig. 1a. As a discharge mediator, the redox potential of RMs needs to be lower than that of Mn^2+^/MnO_2_ (Fig. 1b) and thus can spontaneously reduce solid MnO_2_ during the discharge process, based on Equation (1). Potential candidates with proper redox potential are summarized in Fig. 2a. Given the easy availability, solubility and affordability, the I^–^/I_3_^–^, Br^–^/Br_3_^–^ and Fe^2+^/Fe^3+^ redox couples look promising [37,38].


1 \begin{eqnarray*}
{\mathrm{Mn}}{{{\mathrm{O}}}_{\mathrm{2}}} +{\mathrm{ 4}}{{{\mathrm{H}}}^{\mathrm{ + }}} + {\mathrm{2RM}} &\leftrightarrow &{\mathrm{2}}{{{\mathrm{H}}}_{\mathrm{2}}}{\mathrm{O + M}}{{{\mathrm{n}}}^{{\mathrm{2 + }}}}\\
&&{\mathrm{ + 2R}}{{{\mathrm{M}}}^{\mathrm{ + }}},\Delta {\rm G} < 0 \\
\end{eqnarray*}

In the thermodynamic basis (see φ-pH diagram in Fig. S2a) of acid electrolytes, the oxide potential of Fe^2+^/Fe^3+^ is lower than the equilibrium potential of acid Mn^2+^/MnO_2_, which means the Fe^2+^ can be spontaneously oxidated by the MnO_2_. With the inhibition of hydrolysis in an acidic environment, the oxide trivalent Fe ion exists as soluble Fe^3+^. The UV–vis spectrums and the optical images in Fig. S3 confirm the chemical reaction between Fe^2+^ and MnO_2_ forming Fe^3+^. Those Fe^3+^ ions can be easily electrochemically reduced to Fe^2+^, due to the excellent electrochemical reversibility (Fig. 2b and Fig. S4a). As a result, the solid dead MnO_2_ suspension can be totally reduced to electroactive Mn^2+^ via the Fe^2+^/Fe^3+^ redox mediation (Fig. S4b).

A high electrode potential of the RM can make for the reduction in energy loss caused by a voltage gap (Fig. S5) [28], while leading to a small driving force with sluggish spontaneous chemical reactions. Therefore, the RM reaction kinetics, i.e. the reaction rate between the RM and the dead MnO_2_, needs to be systematically investigated. Compared with I^–^/I_3_^–^ and Br^–^/Br_3_^–^ redox couples (Fig. S6), Fe^2+^ shows the fastest spontaneous chemical reactions with MnO_2_ (Fig. 2c), in which the MnO_2_ powder is completely eliminated within 30 s. In contrast, 120 s is needed to totally eliminate the MnO_2_ by using I^–^ and a very long time of >12 h is needed when using Br^–^. To further quantificationally investigate the RMs reaction kinetics [39–42], their open circuit potential (OCP) changes in the presence of excess MnO_2_ powder are monitored (Fig. 2d and Fig. S7). The Fe^2+^/Fe^3+^ RM possesses the best RM reaction kinetics in comparison with I^–^/I_3_^–^ and Br^–^/Br_3_^–^ RMs (Fig. 2e and Fig. S8). Apparently, the Fe^2+^ is quickly oxidized within 87 s with a transfer coefficient of nearly 100%, which is much faster than that for I^–^ (729 s). As calculated (see details in Supporting Information), the exchange current density (*i*_0_) of the Fe^2+^-mediated MnO_2_ is 6.31 × 10^–11^ A, which is much higher than that of I^–^ (4.26 × 10^–12^ A). Note that Br^–^ hardly works as an efficient RM based on the kinetics analysis. Within 2000 s, Br^–^ is scarcely oxidized by MnO_2_ and its *i*_0_ is as low as 8 × 10^–14^ A. As a result, a huge addition of Br^–^ is necessary to eliminate dead MnO_2_, which may go against high energy density and low cost [28,30]. Hence, the fast reaction kinetics metric of the RM is not only essential for achieving high-rate battery performance, but also crucial for the efficient elimination of dead MnO_2_ with only a little RM addition.

### Insights into the Fe^2+^/Fe^3+^-mediated electrochemical process

**Figure 3.**
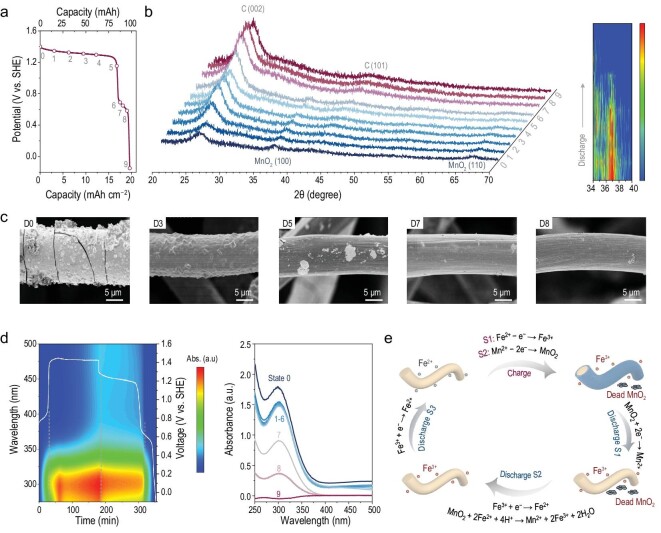
Mechanism and reaction process investigations of Fe^2+^-mediated MnO_2_ cathode. (a) Typical discharge curves with a fixed charge capacity of 20 mAh cm^–2^. (b) *In situ* XRD patterns of MnO_2_ cathode during the discharge process in the 10-mL electrolyte of 0.025 M FeSO_4_ + 1 M MnSO_4_ + 0.5 M H_2_SO_4_ + 1 M Na_2_SO_4_. The right side is the contour map of the MnO_2_ (100) facet. (c) SEM images of Fe–MnO_2_ cathode at different discharge stages. (d) *In situ* UV–vis contour map of the catholyte matching with a galvanostatic charge/discharge curve (left) and selected *in situ* UV–vis spectrum at different discharge stages (right). (e) Schematic illustration of the reaction pathways of the Fe–MnO_2_ cathode during the charge/discharge process.

After establishing the metrics of the RM design and confirming the fast RM reaction, the real effect and mechanism of Fe^2+^ RM during the charge/discharge process of MnO_2_ cathode can be further studied. Figure 3a shows the typical discharge curves of the Fe^2+^-mediated MnO_2_ (Fe–MnO_2_) cathode with a fixed charge capacity of 100 mAh (20 mAh cm^–2^) in 10 mL of the electrolyte for 0.025 M FeSO_4_ + 1 M MnSO_4_ + 0.5 M H_2_SO_4_ + 1 M Na_2_SO_4_. Two discharge plateaus can be observed, i.e. 1.23 V vs. standard hydrogen electrode (SHE) (from D0 to D5 with a capacity of 84.5 mAh) and 0.65 V vs. SHE (from D6 to D8 with a capacity of 13 mAh). Considering the potential of Fe^2+^/Fe^3+^ is 0.77 V vs. SHE, the region from D5 to D9 may relate to the electrochemical reduction of Fe^3+^ to Fe^2+^. Interestingly, in theory, the capacity of the Fe^3+^/Fe^2+^ redox (10 mL of 0.025 M FeSO_4_) is only 6.7 mAh, which is much lower than the observed capacity. This extra capacity is attributed to the regenerative Fe^3+^ from rescuing dead MnO_2_ by the Fe^2+^-mediated reaction.

To figure out the reaction process and charge storage mechanism, the morphological and structural evolution of the Fe–MnO_2_ cathode was investigated by using *ex situ* and *in situ* measurements. New peaks from ε-MnO_2_ appear after the charging process from *in situ* x-ray diffraction (XRD) results (Fig. 3b). In the discharge process from D0 to D5, no diffraction peak shift or new peaks can be observed, only alongside the damping of the ε-MnO_2_ signals, suggesting a MnO_2_ → Mn^2+^ solid/liquid reaction. At D6 (0.68 V vs. SHE), the observable ε-MnO_2_ signals indicate the existence of residual MnO_2_ at the end of the Mn^2+^/MnO_2_ process. Upon further discharging to D7, the MnO_2_ signals disappear, indicating the complete dissolution of dead MnO_2_ with the help of Fe^2+^/Fe^3+^. Scanning electron microscope (SEM) images (Fig. 3c) and x-ray photoelectron spectroscopy (XPS) patterns (Fig. S9) further confirm the structural changes in the electrodes during the discharge process. After charging to 20 mAh cm^–2^ (D0), the surface of the carbon-felt cathode is fully and thickly covered with MnO_2_. During the following discharge, the MnO_2_ layer gradually dissolves but still can be observed at D5 and vanishes in the region of D5–D7.

The mediation effect of Fe^2+^ to eliminate dead MnO_2_ can also be identified by using *in situ* UV–vis spectroscopy (Fig. 3d and Fig. S10). During charge, the generation of Fe^3+^ leads to an increasing absorbance at 296 nm and the subsequent MnO_2_ deposition process accompanied by a continued increase in catholyte absorbance due to the presence of exfoliated MnO_2_. The slight absorbance decrease from D0 to D1 reflects the electro-dissolution of the partially exfoliated MnO_2_. At the discharge states from D1 to D5, the catholyte absorbance remains unchanged but is still higher than the absorbance contributed by Fe^3+^, indicating that the majority of the exfoliated MnO_2_ cannot be discharged and becomes dead MnO_2_. At D6, although the discharge potential comes to the redox reaction of Fe^2+^/Fe^3+^, the Fe^3+^ concentration remains consistent, owing to the regenerated Fe^3+^ from the chemical oxidation of MnO_2_. With the disappearance of MnO_2_ (D6–D8), the Fe^3+^ is gradually consumed and disappears at the end of discharge (D9). Combined with the *in situ* UV–vis contour map results, the whole charge/discharge process of the Fe–MnO_2_ cathode can be summarized as shown in Fig. 3e. The charge process contains two steps, i.e. charge S1, when the Fe^2+^ is electrochemically oxidized to Fe^3+^ first based on Equation (2) due to its relatively low redox potential; charge S2, when the potential is >1.23 V vs. SHE, the electrodeposition of MnO_2_ from Mn^2+^ begins based on Equation (3), with the majority deposited onto the carbon fiber and some dissociated into the electrolyte forming dead MnO_2_:


2 \begin{eqnarray*}
{\mathrm{F}} {{\mathrm{e}}}^{{\mathrm{3 + }}} + {{\mathrm{e}}}^{-} \underset{{\mathrm{charge}}} {\overset{{\mathrm{discharge}}}{\begin{array}{c}\longleftarrow\\
\longrightarrow\end{array}}} {\mathrm{F}} {{\mathrm{e}}}^{{\mathrm{2 + }}} ,\quad{{\mathrm{E}}}_{\mathrm{0}} = 0{\mathrm{.77\, V\, vs}}.\, {\mathrm{SHE}}
\end{eqnarray*}

3 \begin{eqnarray*}
{\mathrm{Mn}}{{{\mathrm{O}}}_{\mathrm{2}}}\,{\mathrm{ +\, 4}}{{{\mathrm{H}}}^{\mathrm{ + }}}\,{\mathrm{ +\, 2}}{{{\mathrm{e}}}^ - }&&\underset{{{\mathrm{Charge}}}}{\overset{{{\mathrm{Discharge}}}}{\begin{array}{c}\longleftarrow\\
\longrightarrow\end{array}}}{\mathrm{2}}{{{\mathrm{H}}}_{\mathrm{2}}}{\mathrm{O + M}}{{{\mathrm{n}}}^{{\mathrm{2 + }}}}{\mathrm{ , }}\\
&&{{{\mathrm{E}}}_{\mathrm{0}}}\,{\mathrm{ = 1}}{\mathrm{.23\, V\, vs}}{\mathrm{.\, SHE}}
\end{eqnarray*}

4 \begin{eqnarray*}
{\mathrm{Mn}}{{{\mathrm{O}}}_{\mathrm{2}}}\,{\mathrm{ +\, 4}}{{{\mathrm{H}}}^{\mathrm{ + }}}\,{\mathrm{ +\, 2F}}{{{\mathrm{e}}}^{{\mathrm{2 + }}}} &\to &{\mathrm{2}}{{{\mathrm{H}}}_{\mathrm{2}}}{\mathrm{O + M}}{{{\mathrm{n}}}^{{\mathrm{2 + }}}}\,{\mathrm{ +\, 2F}}{{{\mathrm{e}}}^{{\mathrm{3 + }}}}{\mathrm{ , }}\\
&&\Delta {{{\mathrm{G}}}^0}\,{\mathrm{ = }} - 89\,\, {\mathrm{ kJ\, mo}}{{{\mathrm{l}}}^{ - {\mathrm{1}}}}
\end{eqnarray*}

The subsequent discharge process contains three steps, i.e. discharge S1, when active MnO_2_ is electrochemically reduced to soluble Mn^2+^; discharge S2, when the potential is <0.77 V vs. SHE, the electrochemical reduction of Fe^3+^ to Fe^2+^ starts, but the generated Fe^2+^ immediately gets chemically oxidated to Fe^3+^ by dead MnO_2_ based on Equation (4); discharge S3, when, after dead MnO_2_ is fully consumed, the Fe^3+^ is gradually reduced to Fe^2+^ until the cut-off potential.

### Device evaluation of Fe^2+^-mediated eZMRFB

**Figure 4.**
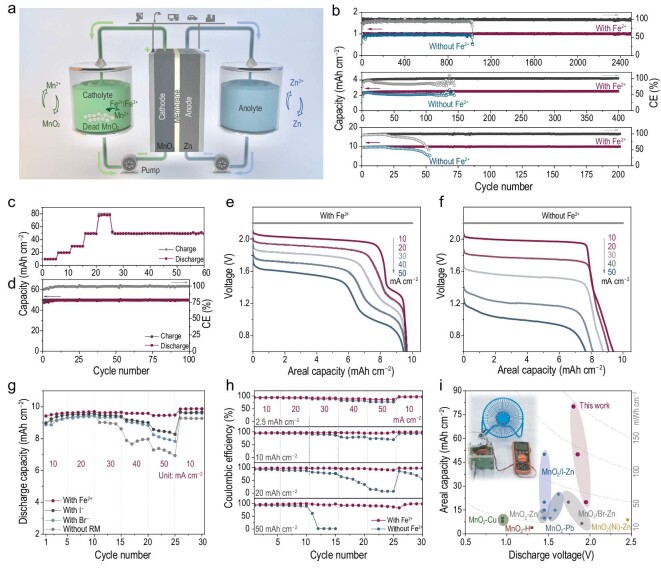
Electrochemical performances of the Fe^2+^-mediated electrolytic Zn–MnO_2_ redox-flow battery. (a) Schematic illustration of the construction of the Fe–eZMRFB. (b) Cycling performance of the cells at 2.5 and 10 mAh cm^–2^ under 20 mA cm^–2^. (c) Cycling performance of the cell at different areal capacities. (d) Long-term cycling at 50 mAh cm^–2^. Discharge curves of the cells (e) with and (f) without Fe^2+^ at different discharge currents. (g) High-rate discharge capacity of different redox mediators with a fixed charge capacity of 10 mAh cm^–2^. (h) High-rate performance with different areal capacities. (i) Comparison of areal capacity and output voltage with other state-of-the-art reported electrolytic MnO_2_ aqueous batteries.

The discharge-mediation role of Fe^2+^/Fe^3+^ for fleetingly and completely eliminating dead MnO_2_ is expected as a potential strategy for practical energy storage with advanced Mn-based aqueous batteries. As a proof of concept, Fe^2+^-mediated electrolytic Zn–MnO_2_ aqueous batteries (Fe–eZMRFB) was fabricated (see illustration in Fig. 4a) by pairing the acidic catholyte of MnSO_4_ + H_2_SO_4_ + Na_2_SO_4_ + FeSO_4_ and the buffered anolyte of ZnSO_4_ + NaAc + HAc (Fig. S11). To minimize the reduced energy density caused by the voltage gap between Fe^2+^/Fe^3+^ and Mn^2+^/MnO_2_ and simultaneously ensure the elimination effect of dead MnO_2_, the addition of Fe^2+^ is controlled at 10% capacity contribution (Fig. S12). With the help of the buffered electrolyte, the Zn anode is protected with enhanced coulombic efficiency (CE) and a stable pH (4.0∼4.8, by *in situ* pH measurement) can be ensured at the Zn anolyte (Figs S13 and S14).

The cycling performance of the eZMRFB cells with and without Fe^2+^ is shown in Fig. 4b. With the discharge mediation of Fe^2+^, the lifespan is >2500 cycles at 1 mAh cm^–2^ with a high CE of nearly 100%. At 2.5 mAh cm^–2^, the initial CE of the Fe^2+^-mediated Zn–MnO_2_ cell is 92.5%, which is much higher than that of the one without Fe^2+^ (74.3%). As a result, the lifespan can be extended from 140 to >400 cycles. Even at a higher areal capacity of 10 mAh cm^–2^, the Fe–eZMRFB still shows stable cycling over 200 cycles, in sharp contrast with the battery without Fe^2+^ (49% capacity retention after 50 cycles). From Fig. S15, the suspended MnO_2_ is observed in the fluid reservoir after a charging process in Fe-free cells, and a good deal of dead MnO_2_ exists in both the electrode and the reservoir. The exfoliated dead MnO_2_ accumulates after cycles, blocking the membrane channel and deteriorating ion transport, thus leading to poor cycling performance. In contrast, the electrolyte remains transparent, indicating much better reversibility via Fe^2+^ discharge mediating. Additionally, Fe^2+^ can also eliminate the potential negative effects of the generation of trivalent Mn species (such as MnOOH and ZnMn_2_O_4_) during the cycling process (Fig. S16). Consequently, the Fe–eZMRFBs with little Fe^2+^ (10 mL of 0.15 M FeSO_4_, which is equivalent to 8.0 mAh cm^–2^) can present 97% CE at a record high capacity of 80 mAh cm^–2^ and capacity utilization of 66.7% (Fig. 4c and Fig. S17). Furthermore, Fe–eZMRFBs support long lifespans at very high areal capacity (>200 cycles at 20 mAh cm^–2^ and >100 cycles at 50 mAh cm^–2^, as seen in Fig. 4d and Fig. S15b).

The high-rate capabilities of eZMRFBs using different RMs with a fixed charge capacity of 10 mAh cm^–2^ are compared in Fig. 4e–g and Fig. S18. The Fe^2+^-mediated cell presents negligible capacity decay (96% of capacity retention) when the discharge current increases from 10 to 50 mA cm^–2^. In contrast, the discharge capacities of the cells without the RM and with I^–^ and Br^–^ RMs are 7.2, 8.3 and 8.0 mAh cm^–2^ at 50 mA cm^–2^, corresponding to capacity retentions of 81.2%, 89.5% and 84.5%, respectively. More importantly, the positive effect of the Fe^2+^ RM is conspicuous in voltage polarization at higher rates, resulting in higher EE. At 50 mA cm^–2^, the cells without the RM and with Fe^2+^, I^–^ and Br^–^ additions present the middle discharge voltages of 0.99, 1.52, 1.05 and 0.91 V, corresponding to EE values of 35%, 63%, 41% and 33%, respectively. The I^–^ and Br^–^ RMs are useful at relatively low rates, but their slow RM reaction kinetics make them inapplicable for high rates. Furthermore, owing to the low solubility, the inevitably generated solid I_2_ and/or liquid Br_2_ would cover the active sites of the electrodeposited MnO_2_ [27,28], hindering the discharge of MnO_2_ and causing large voltage polarization [36]. In contrast, Fe^2+^ shows fast RM reaction kinetics with high solubility of Fe^3+^, which endows the Fe–eZMRFBs with excellent rate capability even at ultra-high areal capacities of >50 mAh cm^–2^ (Fig. 4h).

A comprehensive comparison of the areal capacity and output voltages of Fe–eZMRFBs with other state-of-the-art reported MnO_2_-based aqueous batteries is presented in Fig. 4i and Table S1. With a high areal capacity delivery, the areal energy density of the elaborate Fe–eZMRFBs can reach a record level of 144 mWh cm^–2^, which outperforms most reported aqueous batteries. To further demonstrate the practical potential for large-scale energy-storage applications, an in-series-connected 6-V Fe–eZMRFB stack is assembled to power a 10-W electric fan with a 5.46-V working voltage (Fig. 4i and Fig. S19). The discharge curve of the Fe–eZMRFB stack shows a discharge capacity of 384 mAh, a discharge voltage of 5.68 V, a high CE of 96% and excellent EE of 80.1%. The above results strongly signal that importing Fe^2+^ can boost eZMRFBs for practical application and may immediately benefit reliable and affordable large-scale energy storage.

### Concomitant catalytic acceleration of electrolytic kinetics

**Figure 5.**
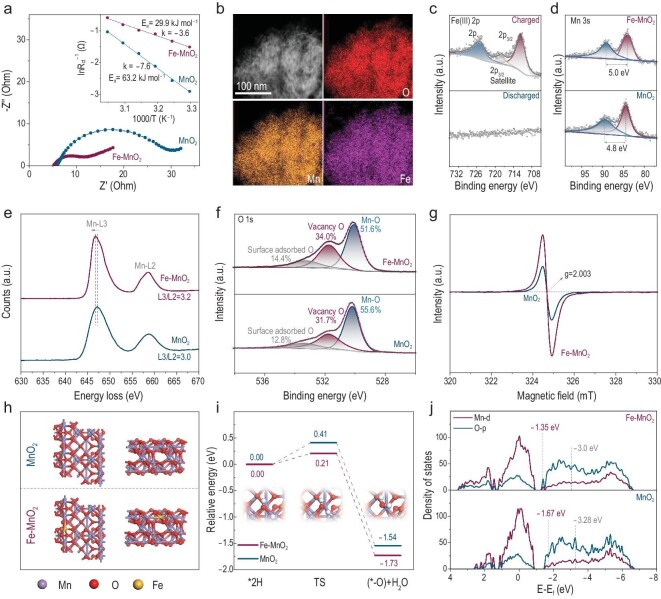
Catalytic acceleration of Fe dopants for electrochemical kinetics of Fe–MnO_2_ and MnO_2_ cathode. (a) EIS spectra. The inset shows the Arrhenius plots of *R*_ct_ values at different temperatures. (b) EDX elemental mapping of Mn, O and Fe in STEM mode. (c) Fe 2p XPS patterns of Fe–MnO_2_ at different charge states. (d) Mn 3s XPS patterns. (e) EELS patterns. (f) O 1s XPS patterns. (g) EPR spectra. (h) Top-view and side-view structures of the electro-oxidized Fe–MnO_2_ (101) and MnO_2_ (101) surfaces. (i) Relative energy profiles with the catalysed electrolysis processes. Insets are schematics of the electro-reaction pathways. (j) PDOS of the O p-band and Mn d-band of Fe–MnO_2_ and MnO_2_ with band center values.

Besides the discharge-mediation effect in eliminating dead MnO_2_, the enhanced reversibility and rate capability of the Fe–MnO_2_ cathode was further revealed. As shown in Fig. S20, the catholyte with Fe^2+^ exhibits a higher current response than the one without Fe^2+^ (pure MnO_2_). In Fig. 5a and Fig. S21, electrochemical impedance spectroscopy (EIS) of the Fe–MnO_2_ cathode shows significantly declined solution and electrode resistance (*R*_b_, 5.2 Ω) compared with pure MnO_2_ (5.7 Ω). The Fe–MnO_2_ cathode exhibits a much smaller charge-transfer resistance (*R*_ct_) value than that of pure MnO_2_ (4.5 Ω for Fe–MnO_2_ vs. 18.4 Ω for pure MnO_2_), implying an increased surface electron mobility and enhanced electrochemical activity in the electrolytic kinetics of Fe–MnO_2_ [20]. Specifically at higher areal capacities, as shown in Fig. S22, the heavy dead MnO_2_ without Fe^2+^ addition leads to an obvious increase in *R*_b_ (from 5.2 Ω at 1 mAh cm^−2^ to 9.7 Ω at 50 mAh cm^−2^) and the *R*_ct_ increases to 130.1 Ω at 50 mAh cm^−2^. For Fe–MnO_2_ at 50 mAh cm^−2^, the *R*_b_ stabilizes at ∼5.4 Ω, indicating an inhibited dead MnO_2_; and *R*_ct_ is only 32.5 Ω. The obtained activation energy (Fig. 5a and Fig. S21a and b) of Fe–MnO_2_ is 29.9 kJ mol^−1^, which is smaller than that of pure MnO_2_ (63.2 kJ mol^−1^). As calculated from the linear polarization curves (Fig. S21c), the interfacial exchange current density for Fe–MnO_2_ (0.92 mA cm^−2^) is also larger than that of pure MnO_2_ (0.48 mA cm^−2^), further indicating accelerated electrolytic kinetics through Fe^2+^ introduction.

By combining high-angle annular dark-field scanning transmission electron microscopy (HADDF-STEM), XPS, electron energy loss spectroscopy (EELS), Raman spectroscopy, electron paramagnetic resonance (EPR) and density functional theory (DFT) calculations, the catalysed electrolytic kinetics of the Fe–MnO_2_ can be identified. The Fe–MnO_2_ shows an extensively exposed nano-leaf morphology with a thickness of 2 nm, with lattice distances corresponding to the facets of ε-MnO_2_ (Fig. S23). The homogeneous distribution of Fe in Mn and O is observed from energy-dispersive X-ray spectroscopy (EDX) elemental mapping (Fig. 5b). The appearance in charge and disappearance after discharge of Fe 2p peaks confirm the doping of Fe ions and high reversibility of the Fe–MnO_2_ cathode (Fig. 5c). The valence state of the Fe dopant is confirmed to be 3+, which is the same as those of reported transition metal ions (e.g. Ni and Co) doped MnO_2_ via electrodeposition [20]. As a result, in Fig. 5d, the enlarged spin-energy splitting (*ΔE*) of Mn 3s doublet peaks (5.0 eV for Fe–MnO_2_ and 4.8 eV for MnO_2_) indicates a reduced valence state of Mn (∼3.3) in Fe–MnO_2_ compared with that of pure MnO_2_ (∼3.6) [7,43,44]. Mn 3s EELS (Fig. 5e) shows the coordination environment of the Mn in Fe–MnO_2_ with the following features: (i) negatively shifted Mn(L_3_) main peak (646.7 eV for Fe–MnO_2_ vs. 647.3 eV for MnO_2_); (ii) higher energy gap between Mn L_2_ and L_3_ edges (11.9 eV for Fe–MnO_2_ vs. 11.5 eV for MnO_2_); and (iii) higher intensity ratio of Mn L_2,3_ edge (3.2 for Fe–MnO_2_ vs. 3.0 for MnO_2_). These results indicate the Mn valence state reduction after Fe^3+^ doping [45,46]. In transition metal oxides, the reduction of metal ions is usually accompanied by the surrounding formation of oxygen vacancy. This means the Fe–MnO_2_ would show an improved oxygen vacancy concentration. In the XPS O 1s profile (Fig. 5f), Fe–MnO_2_ presents a larger oxygen vacancy peak intensity than that of MnO_2_; and Raman results of the Fe–MnO_2_ show an obvious blue shift, and weakening and broadening of the ν_1_ (630−640 cm^−1^) and ν_2_ (550−570 cm^−1^) peaks (Fig. S24). Furthermore, Fe–MnO_2_ possesses an enhanced EPR signal at *g* = 2.003 compared with MnO_2_(Fig. 5g), further confirming the sufficient oxygen vacancies in the Fe–MnO_2_ sample.

As reported, the charge-transfer reaction preferentially proceeds on a potential energy surface with a much lower energy barrier after generating more defects (e.g. Mn and O vacancies) [7,47]. The increased Mn and O vacancies after introducing Fe dopants endow more active electron-transfer kinetics, facilitating electrolytic reaction activity [21,22]. The intrinsic modulation effect of Fe doping on MnO_2_ with catalytic acceleration is further confirmed by the DFT calculations (Fig. 5h–j). Through dissecting the specific reaction pathways during the electrolysis process (Fig. 5i), the energy barrier of defects-rich Fe–MnO_2_ for combining the adsorbed H with the OH group to generate H_2_O is 0.21 eV, which is significantly lower than that of MnO_2_ (0.41 eV). Furthermore, the partial density of states (PDOS) analysis in Fig. 5j indicates that both the Mn d-band center and the O p-band center of Fe–MnO_2_ are obviously upshifted towards the Fermi energy level, which leads to high charge delocalization and active electron states, facilitating charge transfer and thereby catalysing MnO_2_ electrolysis kinetics [21,48]. It can be concluded that the Fe dopants with increased O vacancies are beneficial for lowering the potential energy surface and improving the active O 2p electron states, thus catalysing electrolysis kinetics with lower overpotential.

## CONCLUSIONS

In summary, a bifunctional Fe^2+^ cationic strategy via mediation and catalysis-boosted kinetics is demonstrated to rescue dead MnO_2_ and a stable and fast eZMRFB is constructed. The metrics to all-around evaluate the RM design for eliminating dead MnO_2_ are established. Compared with an anionic RM (e.g. I^–^ and Br^–^), Fe^2+^ is identified to have much faster RM reaction kinetics and more efficient elimination of dead MnO_2_. Combined with *in situ* spectroscopic characterization and electrochemical evaluation, the Fe^2+^/Fe^3+^-mediated electrochemical process and charge storage mechanism are revealed. Meanwhile, as evidenced by electrolytic kinetics, HADDF-STEM, XPS, EELS, EPR and DFT calculations, with intensified oxygen vacancies, Fe-catalysed Mn^2+^/MnO_2_ electrolysis kinetics via charge delocalization and activated O 2p electron states is confirmed. As a proof of concept, the elaborated eZMRFB achieves a coulombic efficiency of nearly 100%, ultra-high areal capacity of 80 mAh cm^–2^, rate capability of 20 C and a long lifespan of 2500 cycles. The results may advance the practical application of energetic aqueous batteries, and benefit reliable and affordable large-scale energy storage.

## Supplementary Material

nwae230_Supplemental_File
