# Supplementary material for: Rescue of dead MnO2 for stable electrolytic Zn–Mn redox-flow battery: a metric of mediated and catalytic kinetics
Source: Natl Sci Rev. 2024 Jul 3;11(8):nwae230. doi: 10.1093/nsr/nwae230 (PMC11312367; doi:10.1093/nsr/nwae230)
Supplement: nwae230_Supplemental_File [file nwae230_supplemental_file.pdf]

SUPPORTING INFORMATION

Rescue dead MnO_2 for stable electrolytic Zn-Mn redox-flow battery: a metric of mediated and catalytic kinetics

Qi Wang[1,†], Wanhai Zhou[1,†,*], Yanyan Zhang[1], Hongrun Jin[1], Xinran Li[1], Tengsheng Zhang[1], Boya Wang[1], Ruizheng Zhao[1], Junwei Zhang[1], Wei Li[1], Yu Qiao[2], Chuankun Jia[3], Dongyuan Zhao[1], and Dongliang Chao[1,*]

[1]Laboratory of Advanced Materials, Shanghai Key Laboratory of Molecular Catalysis and Innovative Materials, State Key Laboratory of Molecular Engineering of Polymers, College of Chemistry and Materials, Fudan University, Shanghai 200433, China;
[2]State Key Laboratory of Physical Chemistry of Solid Surfaces, Collaborative Innovation Center of Chemistry for Energy Materials (iChEM), Department of Chemistry, College of Chemistry and Chemical Engineering, Xiamen University, Xiamen 361005, China;
[3]Institute of Energy Storage Technology, College of Materials Science and Engineering, Changsha University of Science & Technology, Changsha 410114, China
***Corresponding authors.** E-mails: zhouwh@fudan.edu.cn; chaod@fudan.edu.cn
[†]Equally contributed to this work.

Experimental Methods

Chemicals and materials： Zinc sulfate heptahydrate ($ZnSO_4·7H_2O$, AR), manganese sulfate monohydrate ($MnSO_4·H_2O$, AR, 99.0 %), sodium sulfate (Na_2SO_4, AR, 99.0 %), sulfuric acid (H_2SO_4, 95.0–98.0 %), acetic acid (HAc, AR, 99.5%), iron sulfate heptahydrate ($FeSO_4·7H_2O$, AR, ≥ 99.0 %), potassium iodide (KI, AR, ≥ 99.0 %), Potassium bromide (KBr, AR, ≥ 99.0 %) were purchased from Aladdin. All chemicals were used without further purification.

Flow Cell assembly: The Fe^{2+} mediated electrolytic MnO_2-Zn aqueous batteries (Fe-eMnZnABs) were assembled in a purpose-made flow cell. For the full cell, a piece of 0.5 mm zinc plate and a piece of copper foam (1.6 mm) in the anode side. A piece of 5 mm carbon felt was used as cathode and a piece of Nafion® membrane (N117) was applied to avoid ion crossover. The cathodic carbon felt was modified before usage via plasma hydrophilic modification. Before the cell fabrication, the Nafion 117 was firstly treated with H_2O_2 under 80 ℃ for 1h, followed by soaked in 5% H_2SO_4 under 80 ℃ for 1 h. Then, the H^+ type Nafion 117 was treated by 1 M NaOH under 80 ℃ for 2 h, converting to Na^+ type cation exchange membrane. The membrane then was washed by deionized (DI) water and soaked in DI water overnight before usage. The effective geometric electrode area for the flow cell is 5 cm^2 (2 cm × 2.5cm) for both positive and negative electrodes. The buffered electrolyte of NaAc + HAc is used to protect the Zn anode. The H_2SO_4 is added into the catholyte to activate the two-electron reaction of MnO_2. The Na_2SO_4 serves as a supporting electrolyte to balance osmotic pressure and increase the electrolyte conductivity. The anolyte composition is 1 M $ZnSO_4$ + 1 M NaAc + 1 M HAc, and the catholyte composition is 1 M $MnSO_4$ + 0.5 M H_2SO_4 + 1 M Na_2SO_4 + certain amount of $FeSO_4$ determined by practical condition.

Materials Characterizations: The UV-visible (UV-vis) spectra were collected by Shimadzu UV-1800 spectrometer. 1 M $MnSO_4$ aqueous solution was used as the blank electrolyte. X-ray powder diffraction (XRD) patterns were performed by Bruker D8 powder X-ray diffractometer (XRD, Germany) with Cu Kα irradiation (λ = 1.5406 Å). The X-ray photoelectron spectra were obtained by Thermo Fischer ESCALAB 250Xi (America) with Mono Al source (hv = 1486.6 eV) under vacuum condition of $8×10^{-10}$ Pa. The scanning electron microscopy (SEM) images were observed by Zeiss GeminSEM500 (Germany). The high-angle annular dark-field (HAADF) images were acquired with

an ADF detector (annular ranges of 54-220 mrad), dwell times per pixel of 16 μs for 1 k × 1 k image size and 10 μs for 2 k × 2 k image size were used.

Electrochemical measurements: CV, OCP, LSV, and EIS measurements were performed by a VMP3 electrochemical testing unit (Bio-Logic, France), and using a three-electrode configuration composed of a graphite working electrode, a platinum plate counter electrode, and an Ag/AgCl reference electrode. The frequency range of EIS was 0.01-100 kHz with a sine perturbation signal of 5 mV. To compare the electrochemical kinetics of MnO_2 in 1 M $MnSO_4$+0.2 M H_2SO_4 electrolyte and Fe-MnO_2 in 1 M $MnSO_4$+0.2 M H_2SO_4+0.05 M $FeSO_4$ electrolyte, the EIS spectrums of Fe-MnO_2 and MnO_2 with a charge capacity of 5 mAh cm^{-2} were tested at different temperatures. Exchange current density was measured by linear polarization curves (LP) with a charge capacity of 5 mAh cm^{-2}, scanning electrode potential at the rate of 0.1 mV s^{-1} from −5 to 5 mV vs. open circuit potential (OCP).

Kinetic study on the redox mediator reactions

In Fig. 2c, to visually compare the reaction rates of different RM with MnO_2, 50 mg of MnO_2 solid powder was placed in excess of 20 ml 0.1 M $FeSO_4$, 0.15 M KI, and 0.15 M KBr. The supporting electrolyte was 0.5 M H_2SO_4. After stirring at a rate of 300 r min^{-1} for different time periods, optical images were taken to record whether there was solid residue. To investigate the kinetics of the reaction between RMs and MnO_2, the open circuit potential (OCP) changes of RMs in the presence of excess MnO_2 powder is monitored [1-3]. **Fig. 1**e shows the OCP changes of 30 mL solution of 0.1 M $FeSO_4$ + 0.5 M H_2SO_4 upon reacting with 0.5 g MnO_2 (more than 3 times the equivalent capacity of $FeSO_4$). The solution is bubbled by nitrogen gas for half an hour before testing to remove the interference of dissolved oxygen. Note that given the solid material is in considerable excess and kept stirring the solution, the diffusion of Fe^{2+} ion in the solid material would not be the limiting process and the evolution of OCP would solely be dictated by the interfacial charge transfer of the redox mediator reactions. The concentration changes of Fe^{2+} ions are deduced from the OCP of the redox couple in terms of the Nernst equation:

$$E_{OCP} = E^{0}_{\mathrm{Fe^{2+}/Fe^{3+}}} + \frac{RT}{F}\ln\frac{C_{\mathrm{Fe^{3+}}}}{C_{\mathrm{Fe^{2+}}}} \qquad \text{(S1)}$$

where F is the Faraday constant, T is the temperature, R is the universal gas constant, and E_{OCP} and $E^0_{Fe2+/Fe3+}$ are the OCP and standard potential of Fe^{2+}/Fe^{3+}, respectively. Give $\Delta E = E_{OCP} - E^0_{Fe^{2+}/Fe^{3+}}$ and $C_{Fe^{3+}} = 0.1 - C_{Fe^{2+}}$. Then the relationship between ΔE and C can be described as:

$$C_{Fe^{2+}} = \frac{0.1}{e^{\frac{-F\Delta E}{RT}} + 1} \tag{S2}$$

Notice that the standard potential of Fe^{2+}/Fe^{3+} can be measured by CV, that is 0.437 V vs. Ag/AgCl. An effective current can be defined to describe the flux of the RM reaction [4,5]:

$$i_{\text{eff}} = rFV = \frac{\text{d}C_{Fe^{3+}}}{\text{dt}} FV \tag{S3}$$

where V is the electrolyte volume, r is the effective rate of the RM reaction. Since there is a large potential difference between Fe^{2+}/Fe^{3+} and Mn^{2+}/MnO_2, Tafel behavior between i_{eff} and overpotential (η, $\eta = E_{OCP} - E^0_{Mn^{2+}/MnO_2}$) is expected, which allows the determination of the exchange current i_0:

$$\lg i_{\text{eff}} = \lg i_0 - \frac{-\alpha nF}{2.303RT}\eta \tag{S4}$$

Here α is the transfer coefficient. As shown in the inset of **Fig. 1**e, after the system reached a state at which the interfacial charge transfer process becomes the rate-determining step, the linear segment of the lg i_{eff} *vs.* η plot with a slope of $-\alpha F/2.3RT$ could be extrapolated to an intercept of lg i_0. Hence i_0 for Fe^{2+}-MnO_2 is estimated to be around 6.3×10^{-11} A, as α is kept at 1 for a one-way charge transfer process. The kinetics of the reaction between MnO_2 and other RMs (I^- and Br^-) are studied under the same conditions as shown in **Fig. S**7. The Nernst equation handling for iodine and bromine is slightly different from iron. Taking iodine as an example, the relationship between OCP and iodine species concentration can be expressed as follows:

$$E_{OCP} = E^0_{I^-/I_3^-} + \frac{RT}{2F}\ln\frac{C_{I_3^-}}{C_{I^-}^3} \tag{S5}$$

Give $A = \exp(2F(E_{OCP} - E^0)/RT)$, $C = C_{I^-}$, hence $C_{I_3^-} = (0.1 - C)/3$. Then Equation (S3) can be further derived as follows:

$$C^3 - \frac{1}{3A}C - \frac{0.1}{3A} = 0 \tag{S6}$$

The only real solution to this equation, *i.e.*, the concentration of I^-, can be obtained using the root-finding formula for a cubic equation.

DFT Calculations. Density functional theory (DFT) calculations were performed by using the Vienna Ab initio Simulation Package, version of 5.4.4 [6-8], with the projector augmented wave method[4] and a plane wave basis set [9]. The DFT calculations used the generalized gradient approximations of the Perdew–Burke–Ernzerhof functional [10]. The energy cutoff was set to 400 eV. Spin polarization was included in the calculations. The convergence criterion of relaxation for the structural optimizations were a maximum displacement of 0.01 Å. The threshold of self-consistent-field energy convergence was 1.0×10^{-6} eV per atom. Geometric optimizations were used 2×2×1 Monkhorst-Pack grid k-points for the surface (101) and 4×4×2 was sampled for the density of states calculation. A vacuum layer of 12 Å thickness was used in the z-direction to avoid interaction between slabs. The vibrational frequencies were computed to consider the zero-point energies, enthalpy, and entropy and ultimately calculate the free energies at room temperature (298.15 K). The water adsorption energy E_{H2O*} was calculated from $E_{H2O*} = E_{surf+H2O} - E_{surf} - E_{H2O}$, where $E_{surf+H2O}$ and E_{surf} are the total energies of the surface with and without the H_2O adsorbate respectively, and E_{H2O} is the energy of a free water molecule.

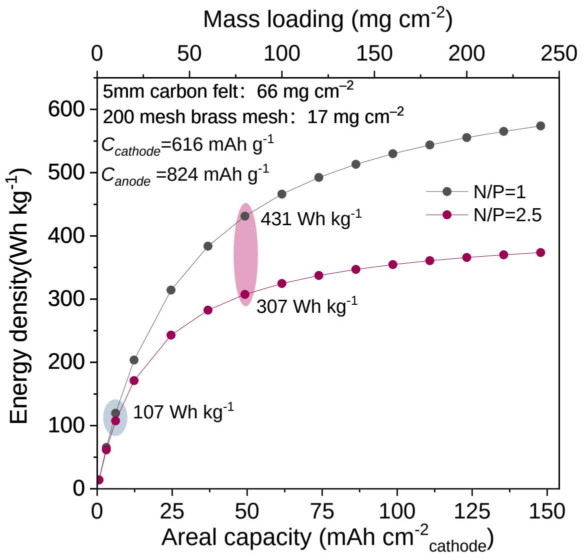

Fig. S1 The relationship between energy density and areal capacity of eMnABs. It is necessary to develop eMnABs with the areal capacity of over 50 mAh cm^{-2} for practical application.

The energy density is calculated based on the total weight of active materials and current collectors of the MnO_2 cathode and zinc anode, according to the following equation:

$$E = \frac{m_C \times C_C \times U}{m_C + m_{CC} + m_{AC} + \frac{m_C \times C_C \times a}{C_A}} \quad (S7)$$

where C_A and C_C correspond to the theoretical capacity of the anode and cathode. m_C is the mass loading of the cathode. m_{CC} and m_{AC} are the weights of the cathodic and anodic current collectors. U is the voltage of the MnO_2-Zn battery (1.95V). a is the value of the N/P ratio. The theoretical capacity of the MnO_2 cathode and zinc anode is 616 mAh g^{-1} and 824 mAh g^{-1}, respectively according to the two-electron reaction. The cathodic current collector is 5 mm carbon felt with a mass of 66 mg cm^{-2}. The anodic current collector is 200 mesh brass mesh with a mass of 17 mg cm^{-2}. When the N/P ratio is 2.5, the energy density of the MnO_2-Zn battery is 307 Wh kg^{-1} with a cathodic mass loading of 80 mg cm^{-2}, i.e., 49.3 mAh cm^{-2}.

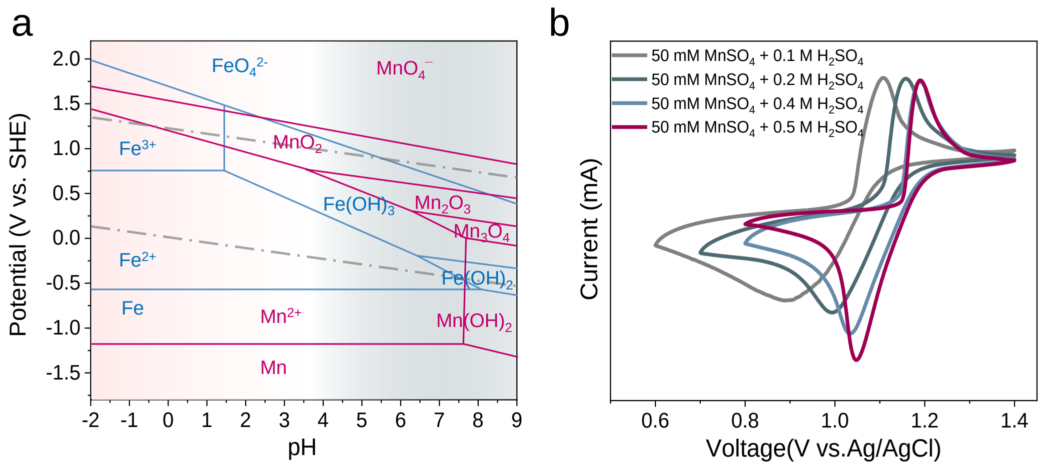

Fig. S2 Feasibility for Fe^{2+}/Fe^{3+} mediator. a) Combined Pourbaix diagram of Fe and Mn. b) CV curves of Mn^{2+}/MnO_2 at different proton concentrations. The acid electrolyte avoids the hydrolysis of Fe^{2+}/Fe^{3+}, and let the Mn^{2+}/MnO_2 electrodeposition process not tend to generate Mn^{3+} species (such as MnOOH). The increase in proton concentration shifts the potential of Mn^{2+}/MnO_2 to more positive values, which is consistent with the Pourbaix diagram.

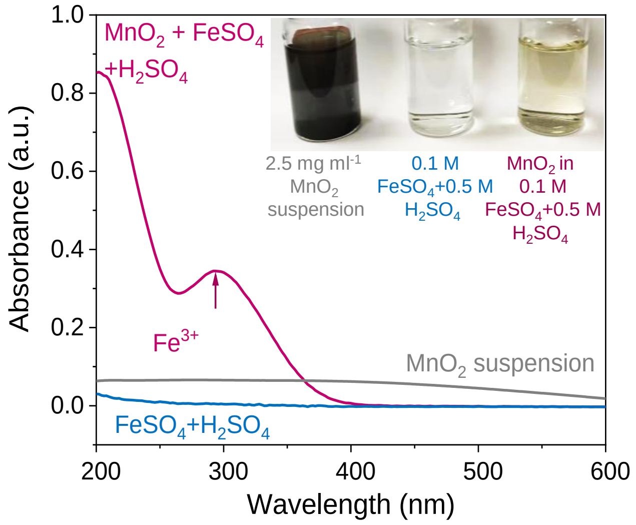

Fig. S3 UV–vis spectrums of 2.5 mg ml^{-1} MnO_2 suspension, 0.1 M $FeSO_4$+ 0.5 M H_2SO_4 solution and the mixture of 50 mg MnO_2 powder + 0.1 M $FeSO_4$ + 0.5 M H_2SO_4 solution. The inset shows the optical images of these samples. The MnO_2 suspension was prepared by sonicating 50 mg MnO_2 powder in 20 ml of water for 10 minutes, and its UV-vis spectrum exhibits broad absorption features. The spectrum of the mixed solution shows a significant absorbance peak at 296 nm, which is consistent with the spectrum of $Fe_2(SO_4)_3$ solution and can be attributed to the generation of Fe^{3+}.

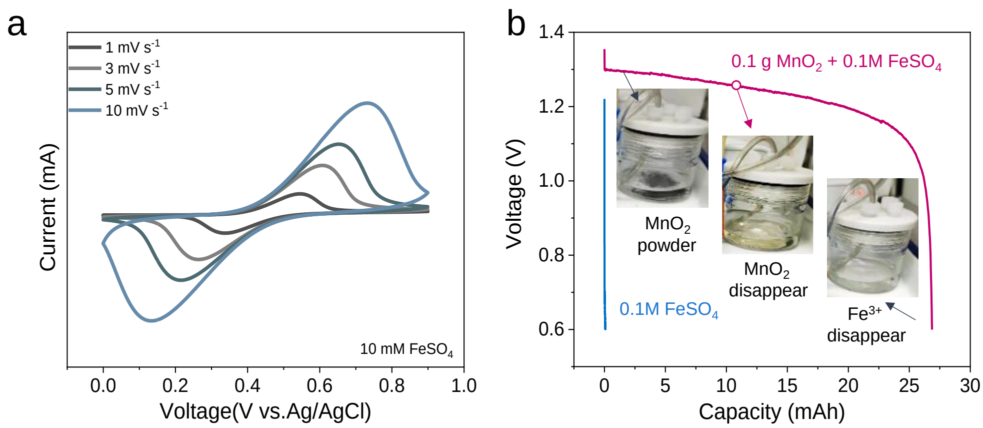

Fig. S4 Fast electrochemical kinetics and reversibility of Fe^{2+}/Fe^{3+}. a) CV curves of 10 mM $FeSO_4$ at different scan rates. b) Discharge profiles of Zn-Fe flow batteries with 20 mL 1 M $Zn(Ac)_2$ as anolyte and with 20 mL 0.1 M $FeSO_4$ + 0.1 g MnO_2 as catholyte. The insets show the optical images of the mixed catholyte during discharge.

The Zn-Fe flow cell is constructed to exhibit the mechanisms of eliminating dead MnO_2 with Fe^{2+} during discharge. Considering Fe^{2+} is reduced state, the $FeSO_4$ catholyte possesses a negligible discharge capacity. After 0.1 g MnO_2 powder is dropped in the cathodic tank, the catholyte starts to change color with a dramatic increase in capacity. Since the mixed catholyte discharge, MnO_2 powder gradually dissolve and the catholyte finally becomes transparent again. The discharge process exhibits 1.25 V *vs.* Zn^{2+}/Zn in a redox flow cell, which is attributed to the reduction of Fe^{3+} to Fe^{2+}. This process demonstrates that the dead MnO_2 can release their capacity without physical contact with the electrode by introducing Fe^{2+}.

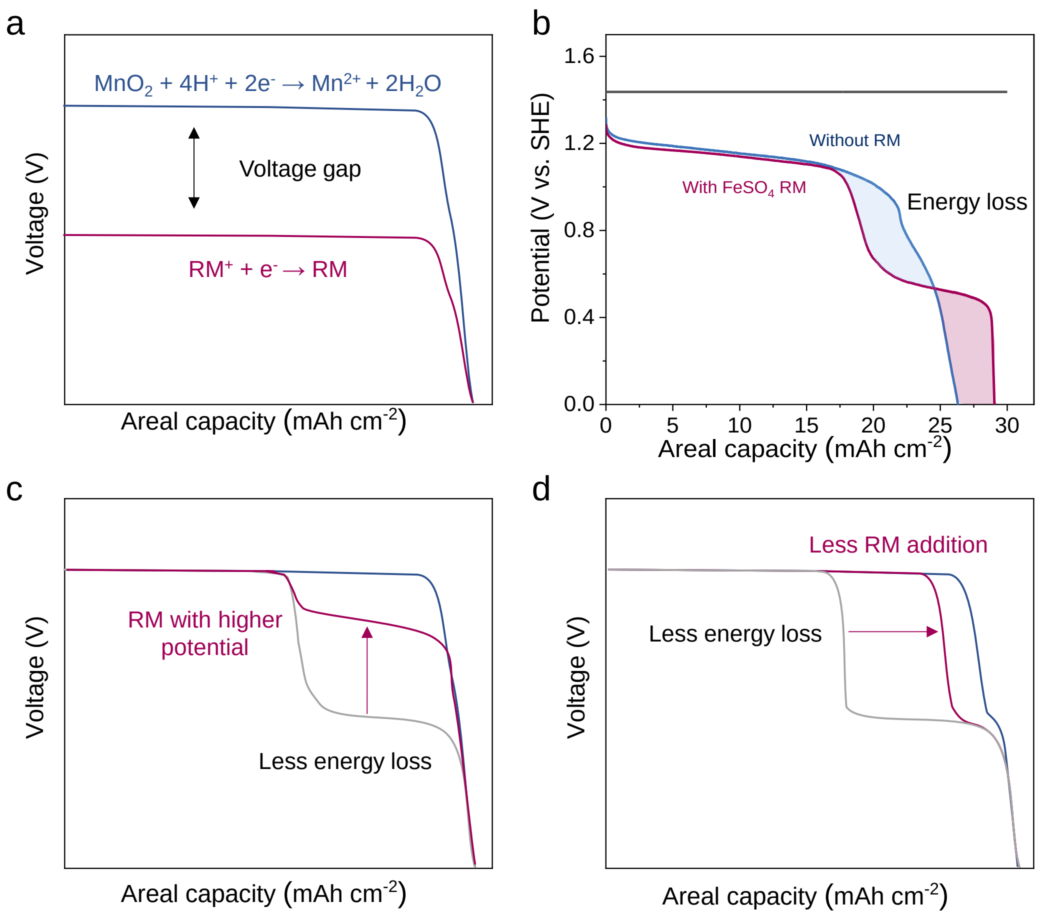

Fig. S5 Schematic of energy loss caused by RM. a) The voltage gap between MnO_2 and RM during discharge. b) Energy losses due to voltage gap under actual working conditions. c, d) Schematic of how to reduce the energy loss.

Since the potential of RM needs to be lower than that of Mn^{2+}/MnO_2, thus with the same total charge capacity, the more RM is introduced, the more energy is lost due to the voltage gap (**Fig. S5**a). During practical operation, while the incorporation of RM enhances the Coulombic efficiency of the battery, the low-grade energy generated during the RM process (the red area in **Fig. S5**b) falls short of the high-grade energy produced during MnO_2 discharge (the blue area in **Fig. S5**b). Especially considering that manganese-based flow batteries have not yet achieved 100% utilization of active materials, it is necessary to impose restrictions on the use of RM to reduce energy loss at the same capacity. Hence, there are two ways to reduce this energy loss: selecting an RM with a potential closer to that of Mn^{2+}/MnO_2, however this means a smaller RM reaction driving force (**Fig. S5**c); the other way is to reduce the amount of RM introduced while ensuring the efficiency of eliminating dead MnO_2, which requires fast reaction kinetics between the RM and the dead MnO_2 (**Fig.S5**d).

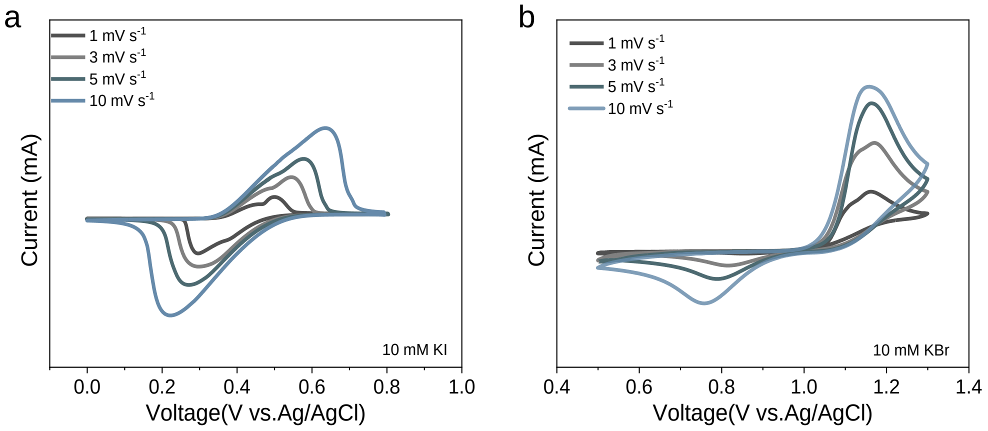

Fig. S6 Electrochemical kinetics and reversibility of I^-/I_3^- and Br^-/Br_3^-. CV curves of a) 10 mM KI in 0.5 M H_2SO_4 and b) 10 mM KBr in 0.5 M H_2SO_4.

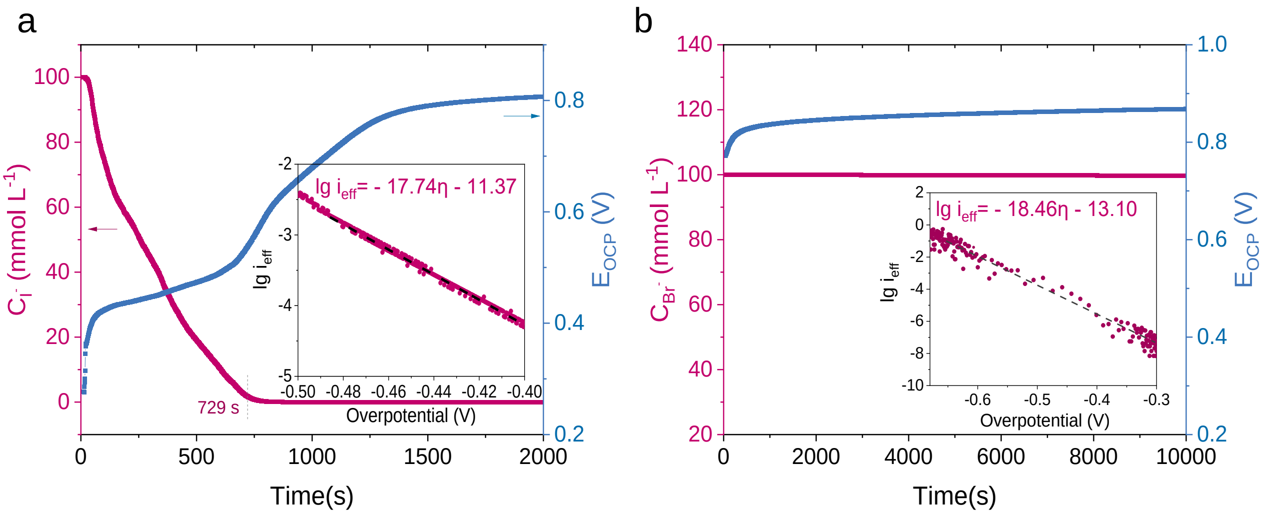

Fig. S7 RM kinetic analysis using iodine and bromine. OCP changes of a) 0.1 M KI + 0.5 M H_2SO_4 and b) 0.1 M KBr + 0.5 M H_2SO_4 solutions upon adding excess 0.5 g MnO_2 powder and the concentration of generated RM^+. The inset shows the changes of reaction flux with overpotential and the fitting in terms of Butler-Volmer formulism.

The exchange current i_0 for I-MnO_2 is estimated to be around 4.26×10^{-12} A， which is lower than that of Fe-MnO_2. For Br-MnO_2, only a small amount of Br^- were reacted and its i_0 is as low as 8×10^{-14} A. The kinetics analysis results are consistent with the observations of the MnO_2 dissolution experiment. Taken together, the intrinsic kinetic superiority of Fe^{2+}-MnO_2 can be verified. Fast RM kinetics will lead to better rate performance of the battery, which is demonstrated in the battery test section. More importantly, fast RM kinetics means that dead MnO_2 can be efficiently eliminate with a low RM addition, thus avoiding the energy loss caused by excessive RM addition and reducing costs.

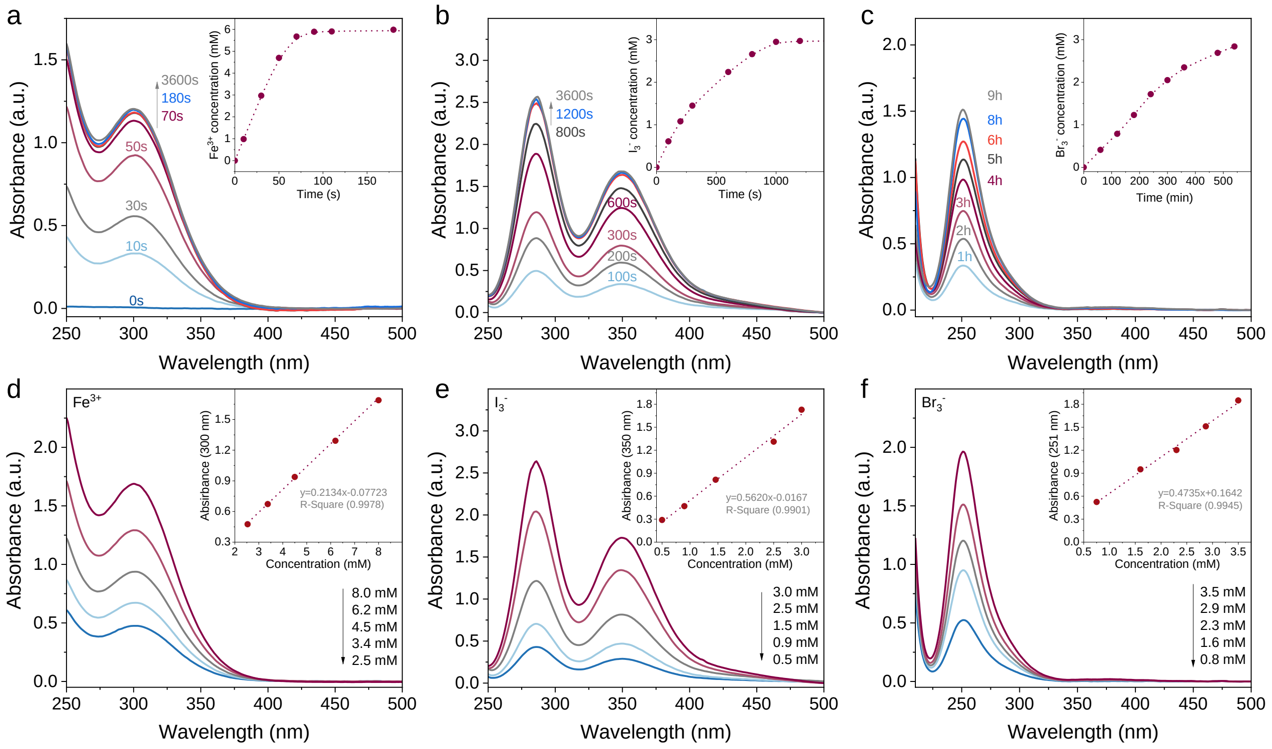

Fig. S8 UV-vis measurements to quantify the changes of concentration of Fe^{3+}, I_3^- and Br_3^- with time during the chemical redox reaction. UV–vis spectrums of a) Fe^{3+}, b) I_3^- and c) Br_3^-. Beer's law plots for d) Fe^{3+} species based on the peak absorbance at 300 nm, e) I_3^- species based on the peak absorbance at 350 nm, and f) Br_3^- species based on the peak absorbance at 251 nm. The fit to the Beer's plots in (d, e and f) were obtained by linear fitting.

UV-vis measurements were conducted using a homemade flowable quartz glass cell with an optical path length of 1 mm. For redox reaction, 3 mg of MnO_2 solid powder was added into 10 ml 6 mM $FeSO_4$, 9 mM KI, and 9 mM KBr with a stirring rate of 50 r min^{-1}. The supporting solution was 0.5 M H_2SO_4. To analyze at a time node, the chemical redox reaction was terminated by fast solvation filtration. The 3.5 mM Br_3^- for standardization is obtained by chemical oxidating of 10 ml 10.5 mM KBr with 3.5 mg MnO_2 and 0.5 M H_2SO_4 at 60 °C with a stirring rate of 1400 r min^{-1} for 2 h.

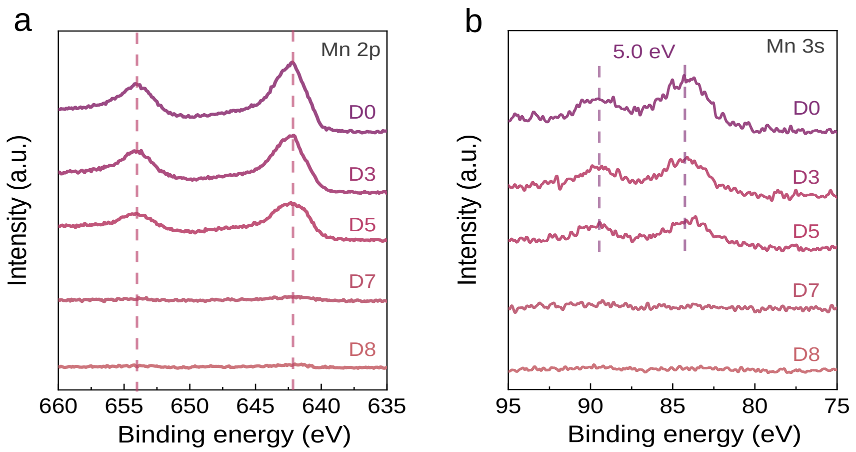

Fig. S9 XPS patterns of Fe-MnO_2 cathode in different states of discharge. a) Mn 2p and b) Mn 3s.

The appearance in charge and disappearance after discharge of Mn 2p and Mn 3s peaks confirm the high-reversibility of deposition and removal of Fe^{2+} mediated Mn^{2+}/MnO_2 cathode. At D0, D3, and D5, the spin-energy splitting (ΔE) of Mn 3s doublet peaks is 5.0 eV, which suggests the average Mn valence state is 3.33 (valence state=8.956–1.126 ΔE) [11-13].

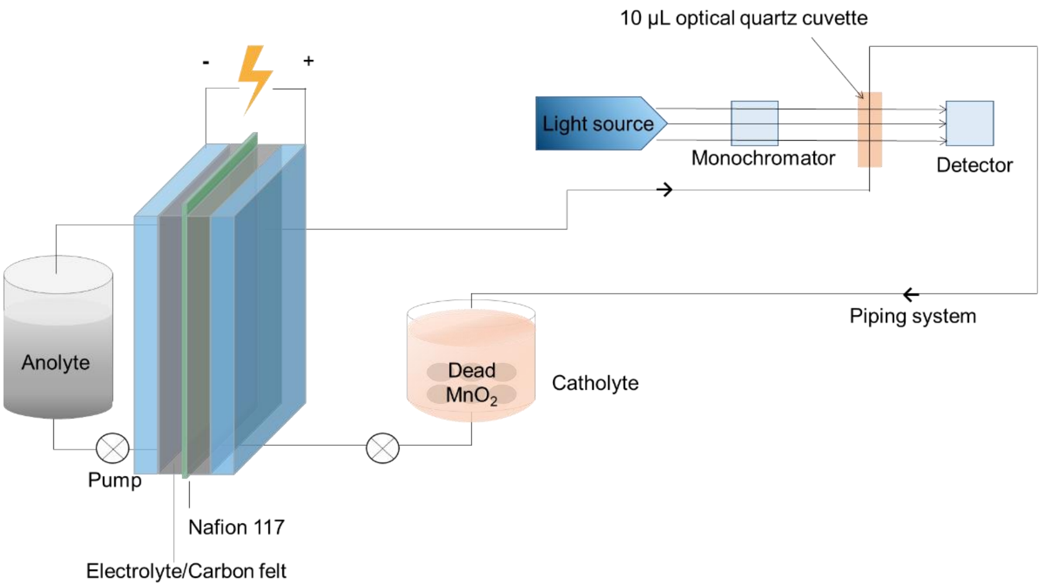

Fig. S10 Configuration of the setup for *in-situ* UV-vis measurement.

For in-situ UV-vis measurement, the catholyte composition is 30 mL 0.006 M $FeSO_4$ + 1 M $MnSO_4$ + 0.5 M H_2SO_4 + 1 M Na_2SO_4 to ensure the absorption of the electrolyte remains within the range of the spectrometer. The charge/discharge process is conducted with a galvanostatic mode of 2 mA cm^{-2} to elucidate the charge storage mechanism.

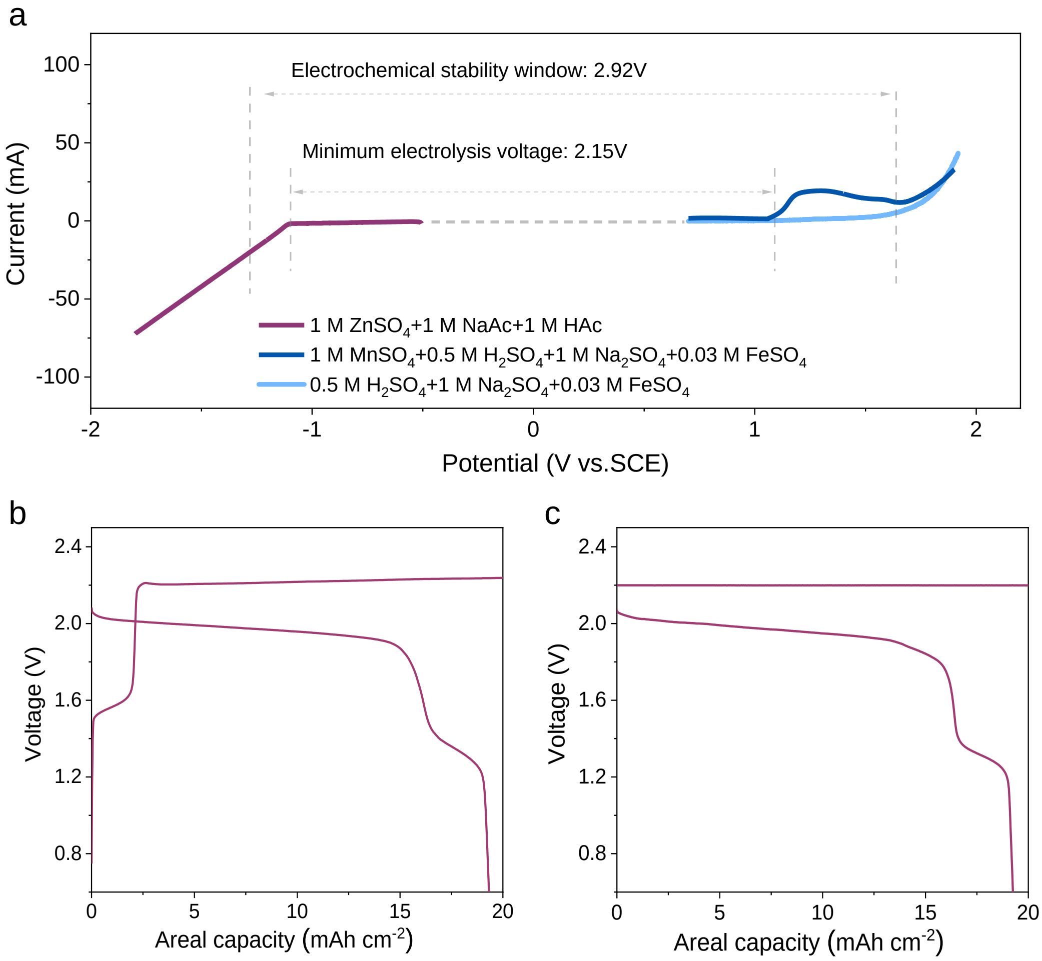

Fig. S11 Design of Fe^{2+}-mediated electrolytic MnO_2-Zn aqueous batteries. a) Verification of the electrochemical stability window (ESW). Voltage curves of Fe-eMnZnABs which are charged at b) galvanostatic mode of 20 mA cm^{-2} and c) constant voltage mode of 2.2 V.

HER and OER properties were obtained from LSV analysis at a scan rate of 5 mV s^{-1} against a saturated SCE reference electrode. These results indicate an ESW of ≈2.92 V in our Fe-eMnZnAB within the potentials of HER and OER. In addition, a minimum electrolysis voltage of ≈2.15 V is required for the simultaneous electrolysis of Zn anode and MnO_2 cathode. The energy efficiency (EE) of the Fe-eMnZnAB is 83% in galvanostatic mode and 78% in constant voltage mode. Although a higher EE can be obtained in galvanostatic charge mode, constant voltage mode ensures the suppression of the oxygen evolution reaction on the cathode during long cycles [12,14,15]. Therefore, based on the electrochemical stability window study, we applied a fixed charge voltage of 2.2 V for Fe-eMnZnAB during long-term cycling.

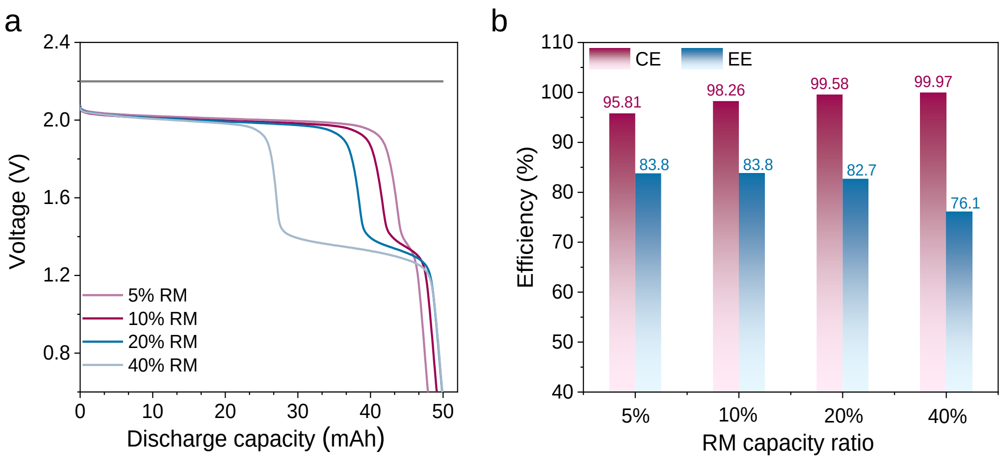

Fig. S12 The influence of the amounts of Fe^{2+} addition. a) Voltage profile and b) efficiencies of Fe-eMnZnAB with different amounts of $FeSO_4$.

It can be seen when more $FeSO_4$ is added, the CE of the cell increases, which means a more adequate RM effect. However, when the addition of $FeSO_4$ reaches to a certain level which is high enough to support the mediation reactions, further increasing the addition of $FeSO_4$ shows limited effect on the CE of the cells but reduces EE due to the voltage gap. Hence, we set the optimal ratio of the equivalent capacity of $FeSO_4$ to 10% of the total charge capacity, to balance the overall battery performance.

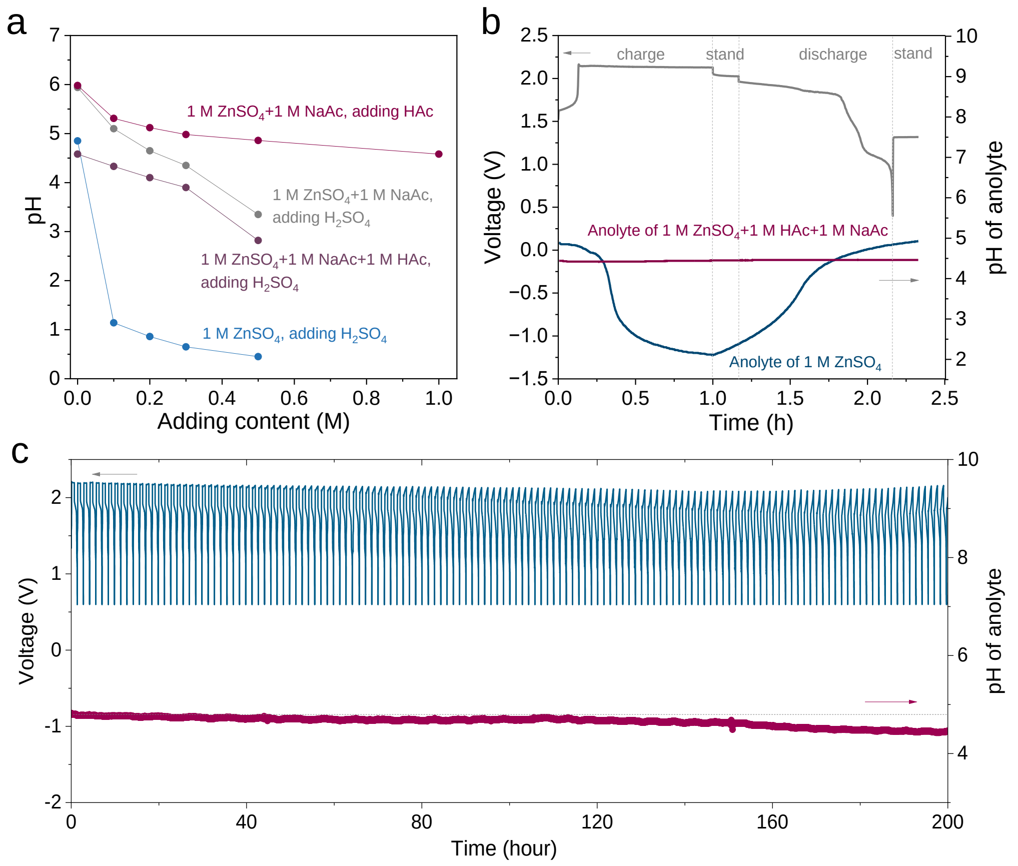

Fig. S13 pH evolution of the anolyte. a) pH changes of anolyte by directly adding acid. b) In-situ pH measurement of Fe-eMnZnABs tested at 10 mAh cm^{-2} using the different anolytes. c) In-situ pH measurement for Fe-eMnZnABs cycling at 10 mAh cm^{-2} using the anolyte of 1 M $ZnSO_4$ + 1 M HAc + 1 M NaAc. The pH of the anolyte remains above 4.0 after more than 200 h of operation. The chemical reaction of Zn species with H^+ species and the electroreduction of H^+ ion on the negative electrode are significantly suppressed at this pH range.

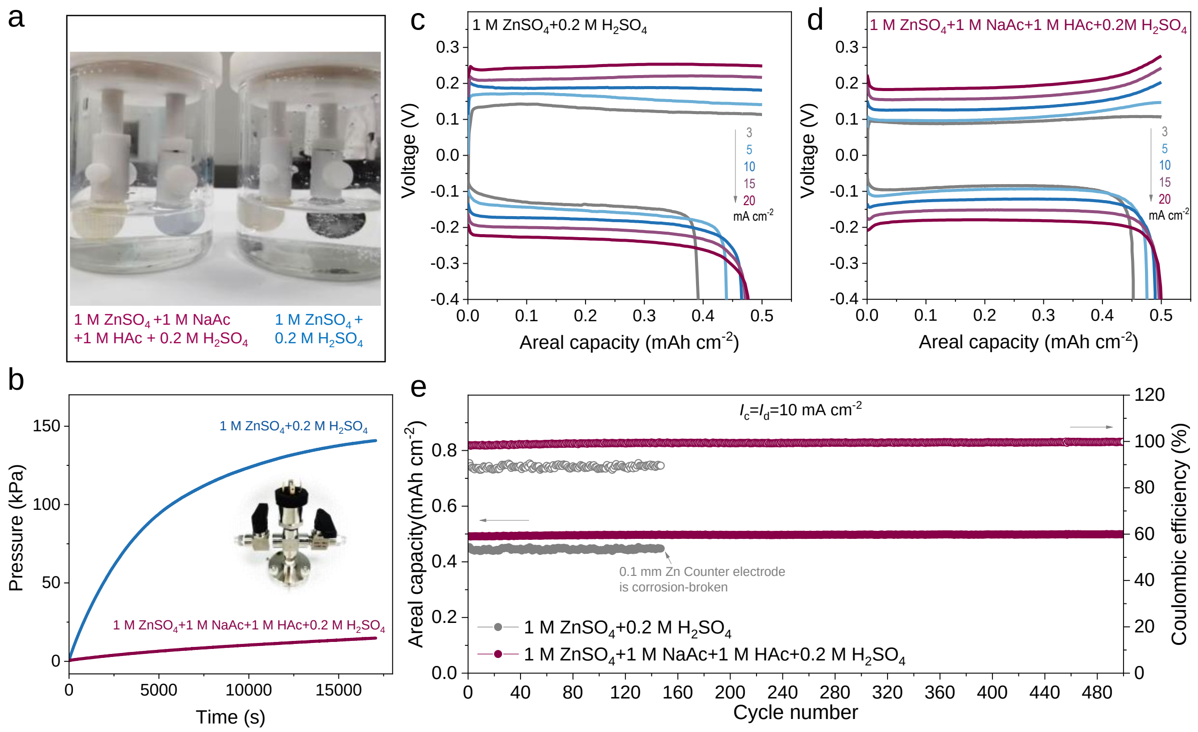

Fig. S14 The stripping/plating performance of Zn anode in buffered electrolyte. a) Optical photographs of the H_2 bubbles on the surface of Zn electrode in an acid electrolyte of 1 M $ZnSO_4$ + 0.2 M H_2SO_4. b) In situ pressure analysis of H_2 production in Zn anode caused by self-corrosion. The plating/stripping curves of Zn/Brass mesh cells with electrolytes of c) 1 M $ZnSO_4$ + 0.2 M H_2SO_4 and d) 1 M $ZnSO_4$ + 1 M HAc +1 M NaAc + 0.2 M H_2SO_4. e) Cycling performance of Zn/Brass mesh cells in different electrolytes.

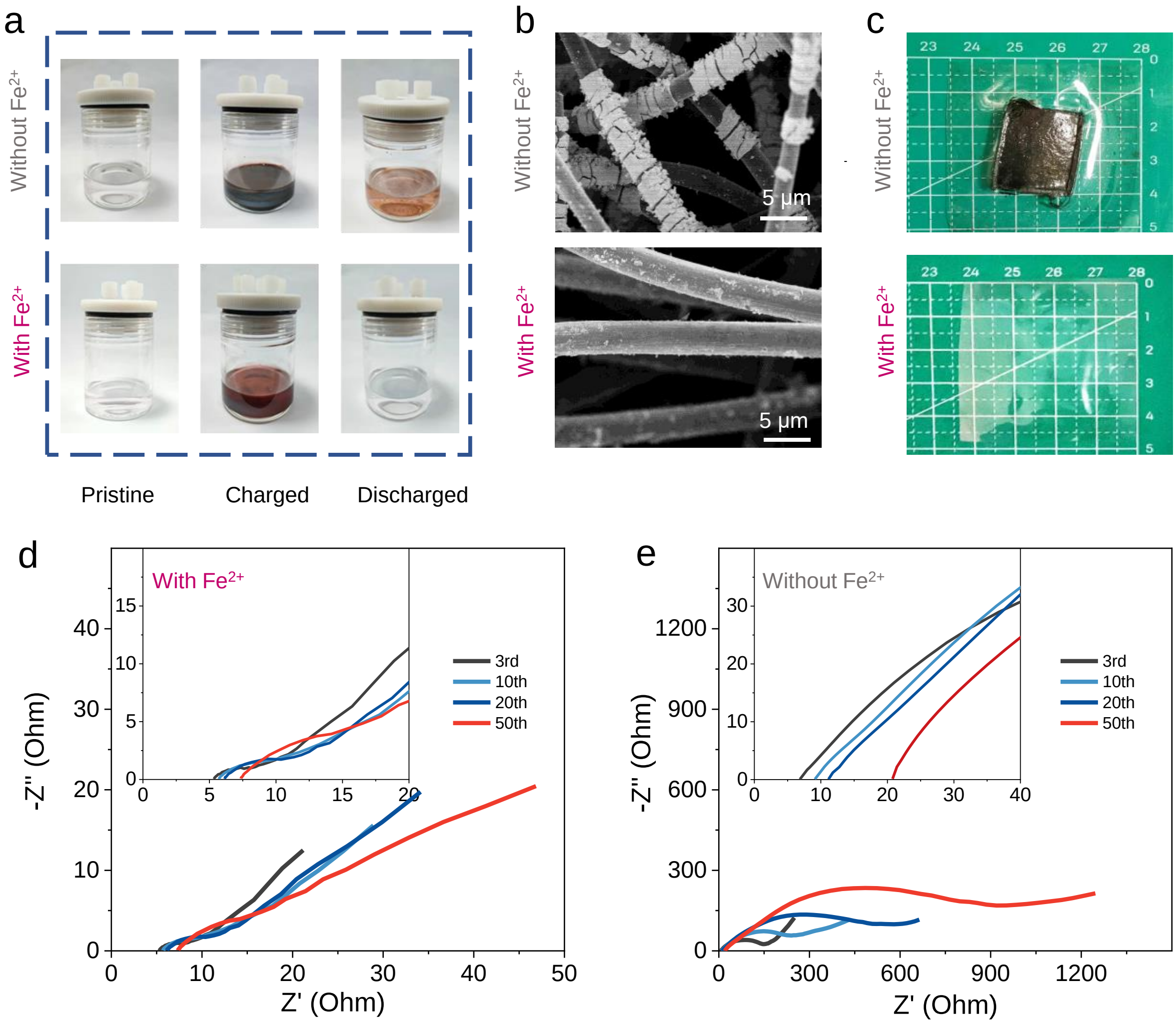

Fig. S15 Comparison of the battery components with Fe^{2+} addition after 20 cycles at 20 mAh cm^{-2}. a) The optical image of the cathodic tank. b) SEM images of cathode carbon felts. c) The optical images of the membranes. EIS spectrums of the cells after different cycles at 10 mAh cm^{-2} d) with and e) without Fe^{2+} addition.

There are obvious suspended particles of MnO_2 in the catholyte after charge. The catholyte with $FeSO_4$ regains clarity after discharge. In contrast, the catholyte without $FeSO_4$ still has dead MnO_2 particles present. These results further support that the $FeSO_4$ mediator effectively dissolves dead MnO_2 and prevents MnO_2 accumulation, thereby improving the cycling stability and CE.

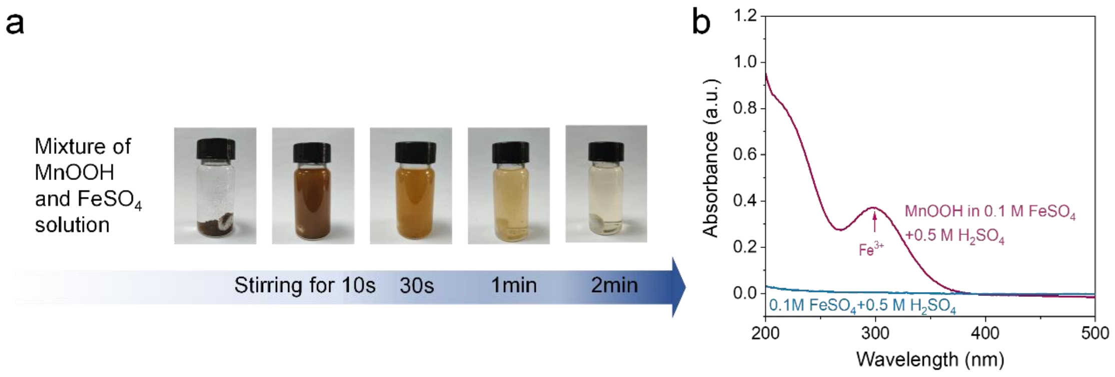

Fig. S16 The reaction process of $FeSO_4$ and MnOOH. a) The optical imagines of the reaction process. b) the UV-Vis spectrum of the reaction products.

20 ml 0.1 M $FeSO_4$ +0.5 M H_2SO_4 solution reacts rapidly with 50 mg MnOOH and the solid dissolves completely when stirred for 2 min. After the reaction, Fe^{3+} was generated in the electrolyte. The results show that Fe^{2+} in an acidic electrolyte can also eliminate potential trivalent Mn species (such as MnOOH and $ZnMn_2O_4$) formed during the cycling.

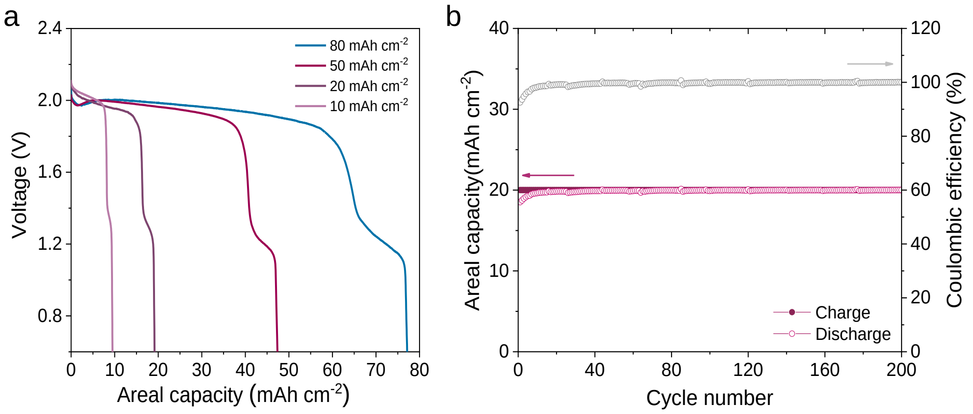

Fig. S17 Electrochemical performances of the Fe-eMnZnABs worked at high areal capacity. a) Discharge curves of Fe-eMnZnABs with different areal capacities. b) The cycling performance of the Fe-eMnZnABs at 20 mAh cm^{-2}. For batteries with different areal capacities, the amount of $FeSO_4$ in each cell is controlled at 10% of the total charge capacity. For example, 10 ml electrolyte of 1 M $MnSO_4$ + 0.5 M H_2SO_4 + 1 M Na_2SO_4 + 0.15 M $FeSO_4$ is used when the areal capacity is 80 mAh cm^{-2} (400 mAh). As a result, the utilization of the catholyte is as high as 66.7%.

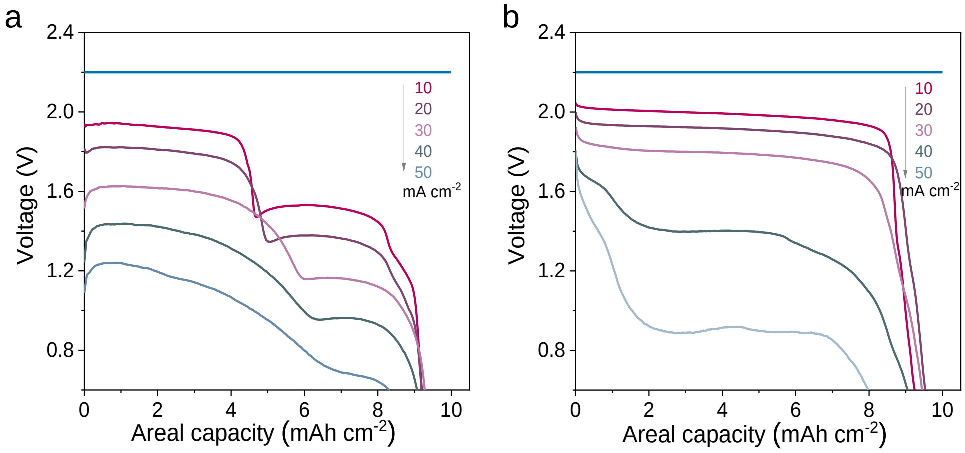

Fig. S18 The rate ability of eMnZnABs with different RMs at 10 mAh cm^{-2}. a) with KI and b) with KBr.

All the RM equivalent areal capacities are 1 mAh cm^{-2}. It can be seen that the performance of eMnZnABs with KI and KBr is limited at high current densities. Decreasing CE at high current densities can be attributed to the slow RM reaction kinetics of I^- and Br^-, resulting in dead MnO_2 not being recovered in time. The potential drop at high rates can be attributed to the insoluble I_2 or Br_2 and accumulated dead MnO_2 cover the active sites of the electrode and thus increases the cell polarization [16,17].

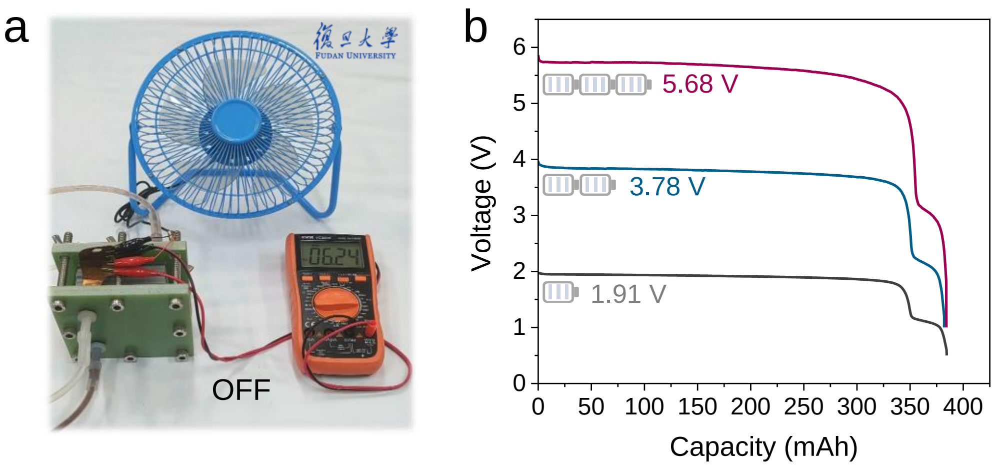

Fig. S19 Practical demonstration of the Fe^{2+} mediated electrolytic MnO_2-Zn aqueous flow battery. a) Digital photograph of a three-in-series cell with a 6.24 V open-circuit voltage. b) Discharge curves of in-series MnO_2-Zn cells.

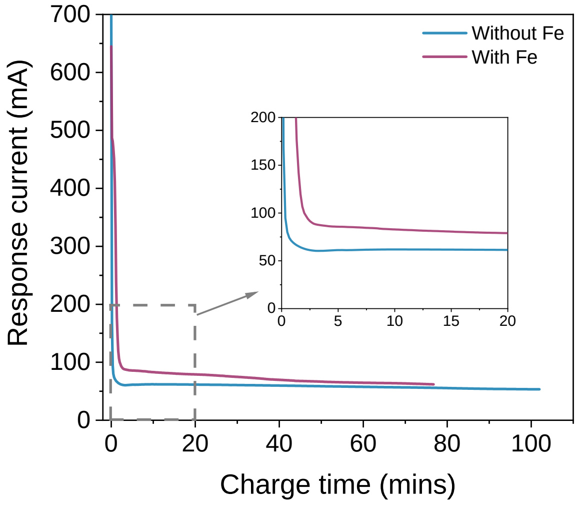

Fig. S20 Current response *vs.* time during chronoamperometry charge.

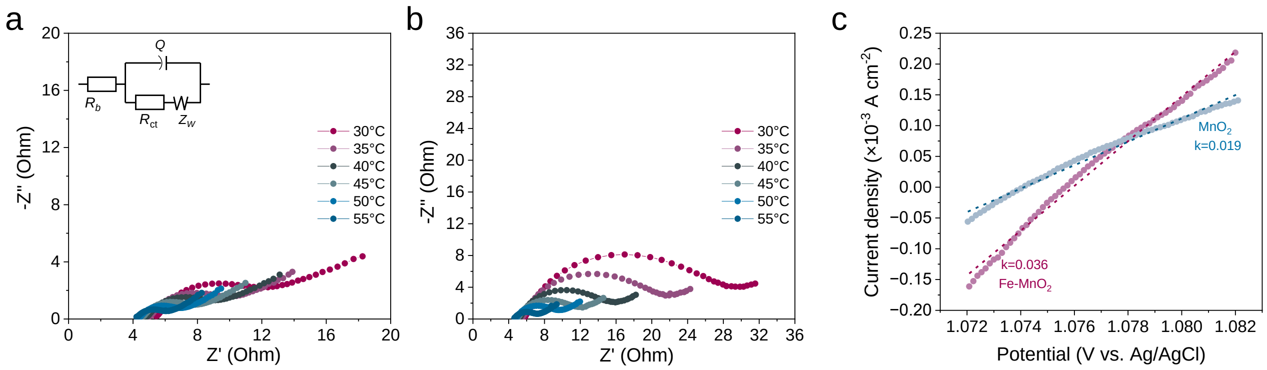

Fig. S21 Electrochemical kinetics of MnO_2 in 1 M $MnSO_4$+0.2 M H_2SO_4 electrolyte and Fe-MnO_2 in 1 M $MnSO_4$ + 0.2 M H_2SO_4 + 0.05 M $FeSO_4$ electrolyte. EIS spectrums of a) Fe-MnO_2 and b) MnO_2 at different temperatures. b) Linear polarization curves.

The activation energy (E_{act}) was calculated by the Arrhenius formula:

$$\frac{1}{R_{ct}} = Aexp(-\frac{E_{act}}{RT}) \tag{S8}$$

where R and T signify the gas constant (8.314 J mol^{-1} K^{-1}) and the temperature (K), respectively.

Exchange current density (I_0) can be calculated from the slopes of linear polarization curves, based on the Equation S9 [18]:

$$I_0 = \frac{I_d RT}{F\eta} \tag{S9}$$

where I_d, F, and η signify the applied current density (A g^{-1}), the Faraday constant (96500 C mol^{-1}) and the total overpotential (V), respectively.

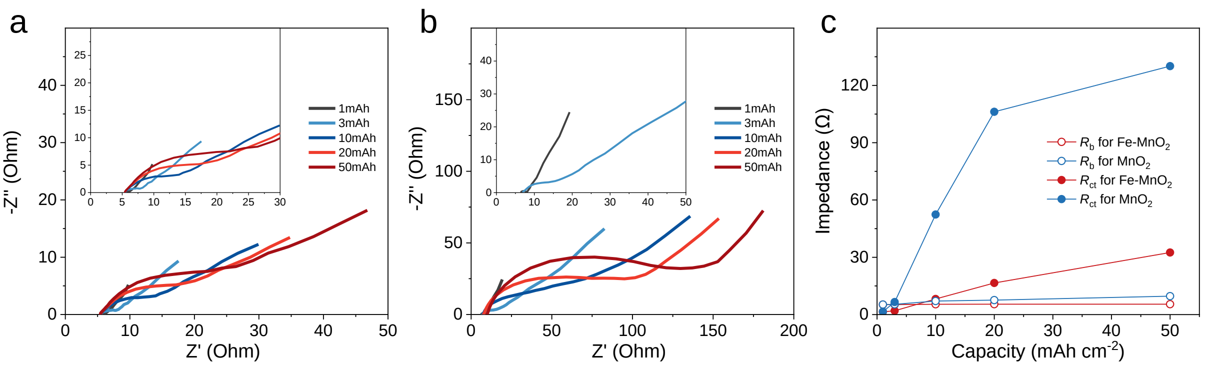

Fig. S22 EIS results at various deposition capacities. a) Fe-MnO_2. b) MnO_2. c) Fitted results of R_b and R_{ct}.

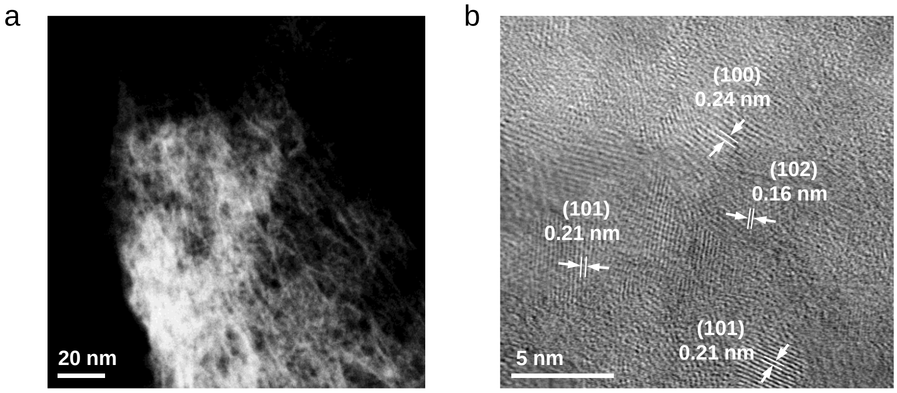

Fig. S23 morphology and crystal structure of the deposited Fe–MnO$_2$. a) HAADF-STEM images. b) High magnification TEM and corresponding FFT pattern. The HADDF-STEM images visualize the nano-leaf morphology of Fe-MnO$_2$ with thickness ≈ 2 nm. The lattice distances of 0.16 nm, 0.21 nm, 0.24 nm correspond to the (102), (101), and (100) facets of ε-MnO$_2$, which is consistent with the XRD results.

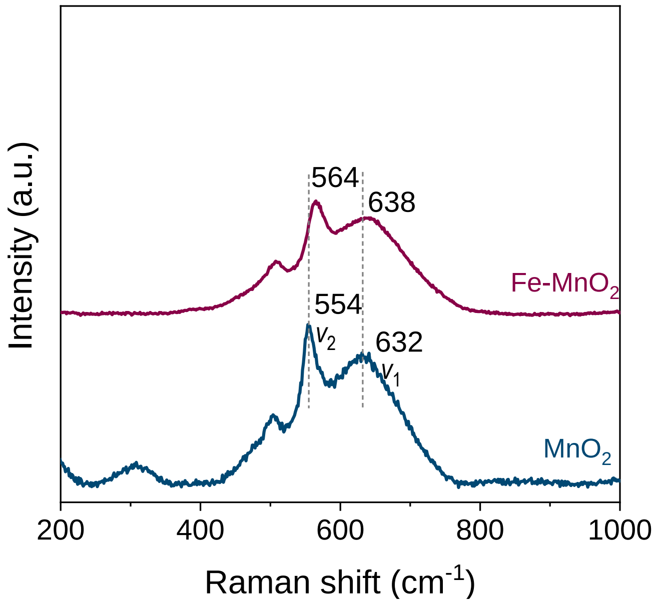

Fig. S24 Raman spectrums of Fe-MnO_2 and MnO_2 cathode

Table S1 Summary of state-of-the-art reported eMnABs.

Battery types	Electrolyte	Catholyte volume (ml)	Max. areal capacity (mAh cm^{-2})	Middle voltage (V)	Rate performance	Cycle performance	Catholyte utilization	Ref.
MnO_2-Zn	1M $MnSO_4$ + 1M Na_2SO_4 + 0.5M H_2SO_4 + x M $FeSO_4$ //1 M $ZnSO_4$ + 1 M NaAc + 1 M HAc	10	80	1.95	99% at 10mA cm^{-2} with 50 mAh cm^{-2}<br>99% at 20 mA cm^{-2} with 50 mAh cm^{-2}<br>90% at 50 mA cm^{-2} with 50 mAh cm^{-2}	2500 cycles at 10 mA cm^{-2} with 1 mAh cm^{-2}<br>200 cycles at 20 mA cm^{-2} with 10 mAh cm^{-2}<br>100 cycles at 20 mA cm^{-2} with 50 mAh cm^{-2}	67%	This work
MnO_2-Zn	1 M $MnSO_4$ + 1 M $ZnSO_4$ + 0.1 M H_2SO_4	1	10	1.95	99% at 2 mA cm^{-2} with 2 mAh cm^{-2}<br>96% at 15 mA cm^{-2} with 2 mAh cm^{-2}<br>87% at 60 mA cm^{-2} with 2 mAh cm^{-2}	1800 cycles at 30 mA cm^{-2} with 2 mAh cm^{-2}	50%	[12]
MnO_2-H_2	1 M $MnSO_4$ + 0.05 M H_2SO_4	0.2	6	1.4	99% at 10 mA cm^{-2} with 1 mAh cm^{-2}<br>98% at 50 mA cm^{-2} with 1 mAh cm^{-2}<br>97% at 100 mA cm^{-2} with 1 mAh cm^{-2}	600 cycles at 10 mA cm^{-2} with 4 mAh cm^{-2}	56%	[15]
MnO_2/I-Zn	1 M Mn(Ac)$_2$ + 1 M Zn(Ac)$_2$ + 2 M KCl + 0.1 M KI	10	50	1.4	95% at 10mA cm^{-2} with 5 mAh cm^{-2}<br>95% at 20 mA cm^{-2} with 5 mAh cm^{-2}<br>94% at 50 mA cm^{-2} with 5 mAh cm^{-2}	400 cycles at 10 mA cm^{-2} with 2.5 mAh cm^{-2}<br>225 cycles at 10 mA cm^{-2} with 20 mAh cm^{-2}<br>50 cycles at 10 mA cm^{-2} with 50 mAh cm^{-2}	36%	[16]
MnO_2/Br-Cd	2 M $MnSO_4$ +1 M HBr+1.5 M H_2SO4+0.5 M $CdSO_4$	11	28	1.55	90% at 40 mA cm^{-2} with 28 mAh cm^{-2}	500 cycles at 80 mA cm^{-2} with 21.4 mAh cm^{-2}<br>150 cycles at 80 mA cm^{-2} with 28 mAh cm^{-2}	68%	[17]
MnO_2-Zn	1 M $MnSO_4$+1 M $ZnSO_4$ + 0.1 M H_2SO_4 + 0.07 mM polyvinylpyrrolidone	/	4	1.9	94% at 4 mA cm^{-2} with 4 mAh cm^{-2}<br>93% at 20 mA cm^{-2} with 4 mAh cm^{-2}<br>88% at 80 mA cm^{-2} with 4 mAh cm^{-2}	2000 cycles at 20 mA cm^{-2} with 4 mAh cm^{-2}	/	[19]
MnO_2-Zn	1.5 M Mn(Ac)$_2$ +1.5 M $ZnCl_2$+ 3 M KCl	50	20	1.4	95% at 20 mA cm^{-2} with 10 mAh cm^{-2}	100 cycles at 10 mA cm^{-2} with 13.3 mAh cm^{-2}<br>50 cycles at 20 mA cm^{-2} with 16.7 mAh cm^{-2}<br>30 cycles at 20 mA cm^{-2} with 20 mAh cm^{-2}	48%	[20]
MnO_2/Br-Zn	1 M $MnSO_4$+1 M $ZnSO_4$ + 0.2 M H_2SO_4 + 0.05 M $ZnBr_2$+ 0.2 M Br_2	25	6.67	1.98	87% at 10 mA cm^{-2} with 6.67 mAh cm^{-2}<br>81% at 26.7mA cm^{-2} with 6.67 mAh cm^{-2}<br>80% at 66.7 mA cm^{-2} with 6.67 mAh cm^{-2}	600 cycles at 6.7mA cm^{-2} with 6.7 mAh cm^{-2}	30%	[21]
MnO_2/I-Zn	1 M $MnSO_4$ + 1 M $ZnSO_4$ + 0.05M KI	18	20	1.85	96% at 10 mA cm^{-2} with 10 mAh cm^{-2}<br>85% at 20 mA cm^{-2} with 10 mAh cm^{-2}<br>80% at 80 mA cm^{-2} with 10 mAh cm^{-2}	180 cycles at 10 mA cm^{-2} with 10 mAh cm^{-2}<br>90 cycles at 20 mA cm^{-2} with 20 mAh cm^{-2}	13%	[22]
MnO_2 (Ni) - Zn	3 M $MnSO_4$ + 0.3 M H_2SO_4 + 0.06M $NiSO_4$ // 3 M NaOH + 0.3 M ZnO	0.5	1	2.5	99% at 2 mA cm^{-2} with 1 mAh cm^{-2}<br>93% at 20 mA cm^{-2} with 1 mAh cm^{-2}<br>88% at 80 mA cm^{-2} with 1 mAh cm^{-2}	600 cycles at 10 mA cm^{-2} with 1 mAh cm^{-2}	30%	[23]
MnO_2-H_2	1 M $MnSO_4$ + 0.1 M H_2SO_4	0.7	20	1.35	95% at 20 mA cm^{-2} with 20 mAh cm^{-2}<br>90% at 50 mA cm^{-2} with 20 mAh cm^{-2}<br>82% at 250 mA cm^{-2} with 20 mAh cm^{-2}	220 cycles at 200 mA cm^{-2} with 20 mAh cm^{-2}	27%	[24]
MnO_2-Zn @Pb	0.2/0.1 M H_2SO_4 + 1 M $ZnSO_4$ + 1 M $MnSO_4$ + 1 mg Pb(OAc)$_2$	5	20	1.95	96% at 4 mA cm^{-2} with 5 mAh cm^{-2}<br>93% at 16 mA cm^{-2} with 5 mAh cm^{-2}<br>92% at 32 mA cm^{-2} with 5 mAh cm^{-2}	800 cycles at 8 mA cm^{-2} with 5 mAh cm^{-2}	25%	[25]
MnO_2-Pb	1 M $MnSO_4$ + 0.5 M H_2SO_4	150	5	1.65	99% at 10 mA cm^{-2} with 1 mAh cm^{-2}<br>99% at 30 mA cm^{-2} with 1 mAh cm^{-2}<br>98% at 100 mA cm^{-2} with 1 mAh cm^{-2}	500 cycles at 5 mA cm^{-2} with 5 mAh cm^{-2}	19%	[26]
MnO_2-Cu	0.3 M $MnSO_4$ + 0.3 M $CuSO_4$ + 0.5 M H_2SO_4	/	3.2	1	95% at 4 mA cm^{-2} with 0.8 mAh cm^{-2}<br>92% at 16 mA cm^{-2} with 0.8 mAh cm^{-2}<br>88% at 64 mA cm^{-2} with 0.8 mAh cm^{-2}	2000 cycles at 16 mA cm^{-2} with 0.8 mAh cm^{-2}	/	[27]
MnO_2-Cu	0.8 M $MnSO_4$ +0.8 M $CuSO_4$ + 0.5 M H_2SO_4	/	50	1.1	98% at 10 mA cm^{-2} with 10 mAh cm^{-2}<br>95% at 100 mA cm^{-2} with 10 mAh cm^{-2}<br>80% at 100 mA cm^{-2} with 50 mAh cm^{-2}	1000 cycles at 10 mA cm^{-2} with 10 mAh cm^{-2}	/	[28]
Mn^{3+}/Fe-Ti	0.1 M $FeSO_4$+1 M $MnSO_4$+1.5 M $TiOSO_4$+3 M H_2SO_4	45/30/15	55	1.2	96% at 40 mA cm^{-2} with 21.2 mAh cm^{-2}<br>97% at 60 mA cm^{-2} with 21.2 mAh cm^{-2}<br>97% at 80 mA cm^{-2} with 21.2 mAh cm^{-2}	500 cycles at 40 mA cm^{-2} with 21.2 mAh cm^{-2}<br>35 cycles at 40 mA cm^{-2} with 55 mAh cm^{-2}	78%	[29]
MnO_2/I-S	1.5 M Mn(Ac)$_2$ + 2 M KAc + 0.2 M KI +1.5 M KCl	10	100	0.98	98% at 40 mA cm^{-2} with 10 mAh cm^{-2}	75 cycles at 10 mA cm^{-2} with 50 mAh cm^{-2}<br>25 cycles at 10 mA cm^{-2} with 100 mAh cm^{-2}	47%	[30]
MnO_2/Fe-Cu	0.001 M $FeSO_4$ + 0.4 M $MnSO_4$ + 0.4 M $CuSO_4$ + 0.5 M H_2SO_4	1	2	/	98% at 3 mA cm^{-2} with 2 mAh cm^{-2}	200 cycles at 3 mA cm^{-2} with 2 mAh cm^{-2}	15%	[31]
MnO_2-Zn	1 M $MnSO_4$ + 1 M $ZnSO_4$	520	2.2	1.78	99% at 2 mA cm^{-2} with 0.5 mAh cm^{-2}	500 cycles at 2.2 mA cm^{-2} with 2.2 mAh cm^{-2}	4%	[32]

Reference

1. Zhang F, Gao M, Huang S *et al.* Redox targeting of energy materials for energy storage and conversion. *Adv Mater* 2022; **34**: 2104562.
2. Zhou M, Chen Y, Zhang Q *et al.* $Na_3V_2(PO_4)_3$ as the sole solid energy storage material for redox flow sodium-ion battery. *Adv Energy Mater* 2019; **9**: 1901188.
3. Zhang H, Zhang F, Yu J *et al.* Redox targeting-based thermally regenerative electrochemical cycle flow cell for enhanced low-grade heat harnessing. *Adv Mater* 2021; **33**: e2006234.
4. Huang S, Yuan Z, Salla M *et al.* A redox-mediated zinc electrode for ultra-robust deep-cycle redox flow batteries. *Energy Environ Sci* 2023; **16**: 438-445.
5. Huang S, Zhang H, Zhuang J *et al.* Redox-Mediated Two-Electron Oxygen Reduction Reaction with Ultrafast Kinetics for Zn-Air Flow Battery. *Adv Energy Mater* 2022; **12**: 2103622.
6. Kresse G, Hafner J. Ab initio molecular dynamics for liquid metals. *Phys Rev B* 1993; **47**: 558-561.
7. Kresse G, Furthmüller J. Efficiency of ab-initio total energy calculations for metals and semiconductors using a plane-wave basis set. *Computational Materials Science* 1996; **6**: 15-50.
8. Kresse G, Furthmüller J. Efficient iterative schemes for ab initio total-energy calculations using a plane-wave basis set. *Phys Rev B* 1996; **54**: 11169-11186.
9. Kresse G, Joubert D. From ultrasoft pseudopotentials to the projector augmented-wave method. *Phys Rev B* 1999; **59**: 1758-1775.
10. Perdew JP, Burke K, Ernzerhof M. Generalized Gradient Approximation Made Simple. *Physical Review Letters* 1996; **77**: 3865-3868.
11. Chigane M, Ishikawa M. Manganese oxide thin film preparation by potentiostatic electrolyses and electrochromism. *J Electrochem Soc* 2000; **147**: 2246.
12. Chao D, Zhou W, Ye C *et al.* An Electrolytic Zn–MnO_2 Battery for High-Voltage and Scalable Energy Storage. *Angew Chem Int Ed* 2019; **58**: 7823-7828.
13. Galakhov VR, Demeter M, Bartkowski S *et al.* Mn3s exchange splitting in mixed-valence manganites. *Phys Rev B* 2002; **65**: 113102.
14. Liu C, Chi X, Han Q *et al.* A High Energy Density Aqueous Battery Achieved by Dual Dissolution/Deposition Reactions Separated in Acid-Alkaline Electrolyte. *Adv Energy Mater* 2020; **10**: 1903589.
15. Chen W, Li G, Pei A *et al.* A manganese–hydrogen battery with potential for grid-scale energy storage. *Nat Energy* 2018; **3**: 428-435.
16. Lei J, Yao Y, Wang Z *et al.* Towards high-areal-capacity aqueous zinc–manganese batteries: promoting MnO_2 dissolution by redox mediators. *Energy Environ Sci* 2021; **14**: 4418-4426.
17. Liu Y, Xie C, Li X. Bromine assisted MnO_2 dissolution chemistry: toward a hybrid flow battery with energy density of over 300 Wh L^{-1}. *Angew Chem Int Ed* 2022; **61**: e202213751.
18. Notten P, Hokkeling P. Double-phase hydride forming compounds: a new class of highly electrocatalytic materials. *J Electrochem Soc* 1991; **138**: 1877.
19. Chuai M, Yang J, Tan R *et al.* Theory-Driven Design of a Cationic Accelerator for High-Performance Electrolytic MnO_2–Zn Batteries. *Adv Mater* 2022; **34**: 2203249.
20. Xie C, Li T, Deng C *et al.* A highly reversible neutral zinc/manganese battery for stationary energy storage. *Energy Environ Sci* 2020; **13**: 135-143.

21. Zheng X, Wang Y, Xu Y *et al.* Boosting Electrolytic MnO_2–Zn Batteries by a Bromine Mediator. *Nano letters* 2021; **21**: 8863-8871.
22. Zheng X, Luo R, Ahmad T *et al.* Development of high areal capacity electrolytic MnO_2-Zn battery via an iodine mediator. *Energy Environ Mater* 2023; **6**: e12433.
23. Chao D, Ye C, Xie F *et al.* Atomic engineering catalyzed MnO_2 electrolysis kinetics for a hybrid aqueous battery with high power and energy density. *Adv Mater* 2020; **32**: 2001894.
24. Xiao X, Zhang Z, Wu Y *et al.* Ultrahigh-Loading Manganese-Based Electrodes for Aqueous Batteries via Polymorph Tuning. *Adv Mater* 2023; **35**: 2211555.
25. Ruan P, Chen X, Qin L *et al.* Achieving Highly Proton-Resistant Zn–Pb Anode through Low Hydrogen Affinity and Strong Bonding for Long-Life Electrolytic Zn//MnO_2 Battery. *Adv Mater* 2023; **35**: 2300577.
26. Huang J, Yan L, Bin D *et al.* An aqueous manganese–lead battery for large-scale energy storage. *J Mater Chem A* 2020; **8**: 5959-5967.
27. Liang G, Mo F, Li H *et al.* A Universal Principle to Design Reversible Aqueous Batteries Based on Deposition–Dissolution Mechanism. *Adv Energy Mater* 2019; **9**: 1901838.
28. Huang JH, Guo ZW, Dong XL *et al.* Low-cost and high safe manganese-based aqueous battery for grid energy storage and conversion. *Sci Bull* 2019; **64**: 1780-1787.
29. Nan M, Wu M, Liu Y *et al.* Boosting the Areal Capacity of Titanium-Manganese Single Flow Battery by Fe^{2+}/Fe^{3+} Redox Mediator. *Small Methods* 2023; **7**: e2201266.
30. Lei J, Yao Y, Huang Y *et al.* A highly reversible low-cost aqueous sulfur–manganese redox flow battery. *ACS Energy Lett* 2022; **8**: 429-435.
31. Ye X, Han D, Jiang G *et al.* Unraveling the deposition/dissolution chemistry of MnO_2 for high-energy aqueous batteries. *Energy Environ Sci* 2023; **16**: 1016-1023.
32. Li GD, Chen W, Zhang H *et al.* Membrane-Free Zn/MnO_2 Flow Battery for Large-Scale Energy Storage. *Adv Energy Mater* 2020; **10**: 1902085.
